# Phosphodiester models for cleavage of nucleic acids

**DOI:** 10.3762/bjoc.14.68

**Published:** 2018-04-10

**Authors:** Satu Mikkola, Tuomas Lönnberg, Harri Lönnberg

**Affiliations:** 1 Department of Chemistry, University of Turku, FIN-20014 Turku, Finland

**Keywords:** Cleavage, DNA, kinetics, mechanism, RNA

## Abstract

Nucleic acids that store and transfer biological information are polymeric diesters of phosphoric acid. Cleavage of the phosphodiester linkages by protein enzymes, nucleases, is one of the underlying biological processes. The remarkable catalytic efficiency of nucleases, together with the ability of ribonucleic acids to serve sometimes as nucleases, has made the cleavage of phosphodiesters a subject of intensive mechanistic studies. In addition to studies of nucleases by pH-rate dependency, X-ray crystallography, amino acid/nucleotide substitution and computational approaches, experimental and theoretical studies with small molecular model compounds still play a role. With small molecules, the importance of various elementary processes, such as proton transfer and metal ion binding, for stabilization of transition states may be elucidated and systematic variation of the basicity of the entering or departing nucleophile enables determination of the position of the transition state on the reaction coordinate. Such data is important on analyzing enzyme mechanisms based on synergistic participation of several catalytic entities. Many nucleases are metalloenzymes and small molecular models offer an excellent tool to construct models for their catalytic centers. The present review tends to be an up to date summary of what has been achieved by mechanistic studies with small molecular phosphodiesters.

## Introduction

**Figure 1:**
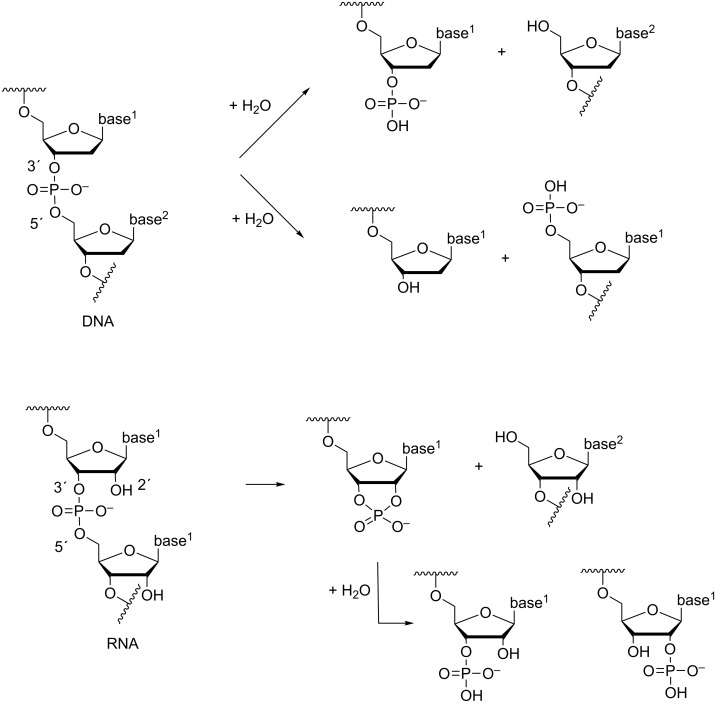
Enzymatic cleavage of phosphodiester linkages of DNA and RNA.

Nucleic acids are polymeric diesters of phosphoric acid that store and transfer biological information. In biological systems, the diester linkages bridging 3´-O of one nucleoside to the 5´-O of the next one are cleaved by a variety of enzymes [1]. The phosphodiester bonds of DNA are hydrolyzed, depending on the enzyme, either to a 3´- or 5´-phosphate, whereas the bonds in RNA, with few exceptions (above all RNase H-catalyzed cleavages) undergo transesterification to a 2´,3´-cyclic phosphate that is rapidly hydrolyzed to 2´- and 3´-phosphates (Figure 1). In the absence of any catalyst, the 3´,5´-phosphodiester linkages are remarkably stable under physiological conditions. The half-life for the hydrolysis of an individual phosphodiester bond in DNA has been estimated to be 30 million years at 25 °C, which means that protein enzymes, nucleases, are able to accelerate the phosphodiester cleavage by a factor of 10^17^ [2]. The phosphodiester linkages of RNA are much more labile, owing to the presence of neighboring hydroxy function that serves as an intramolecular nucleophile resulting in transphosphorylation by departure of the 5´-linked nucleoside [3]. The half-life at pH 6–7 and 25 °C is around 10 years [4–5], the enzymatic cleavage by RNase A being 3∙10^11^ times faster [6]. Interestingly, the RNA phosphodiester bonds are additionally subject to cleavage by RNA itself, viz. by RNA sequences known as ribozymes [7]. The length of these catalytic sequences varies from 70–150 nucleotides of the so-called small ribozymes to hundreds of nucleotides of large ribozymes. Their catalytic efficiency is somewhat more modest than that of protein enzymes.

The remarkable catalytic efficiency has made the action of protein nucleases and ribozymes a subject of intensive mechanistic studies. pH-Rate dependency, X-ray structures, amino acid/nucleotide substitution experiments and the effect of thiosubstitution of phosphate oxygens on the binding of metal ion cofactors have given invaluable information about the residues that participate in substrate binding or contribute to formation of high-energy intermediates or transition states during the PO-bond cleavage by protein nucleases [8] or ribozymes [9–10]. Based on this data, energetics of various pathways from the reactants to products may be compared by computational methods [11–14]. Still, experimental studies with small molecular model compounds play an essential role in mechanistic studies of the enzymatic cleavage of nucleic acids. With small molecules, the importance of various elementary processes, such as proton transfer and metal ion binding, for stabilization of transition states may be elucidated and systematic variation of the basicity of the entering and departing nucleophile enables determination of the position of the transition state on the reaction coordinate. Such data is important on analyzing enzyme mechanisms based on synergistic participation of several catalytic entities. Similar studies are not possible with enzymes, since even a minor change in the structure of enzyme or substrate may have a dramatic effect on the structure and stability of the enzyme–substrate complex. In addition, the kinetic data obtained with small molecules is useful for testing the validity of computational methods utilized for the generation of energy landscapes for enzyme catalysis [15–17].

Many nucleases are metalloenzymes containing two catalytically active metal ions. Small molecular models offer an excellent tool to study the cooperative action of metal ions and to construct models for catalytic centers [11,18].

## Review

### Basic principles of phosphoryl transfer reactions

**Figure 2:**
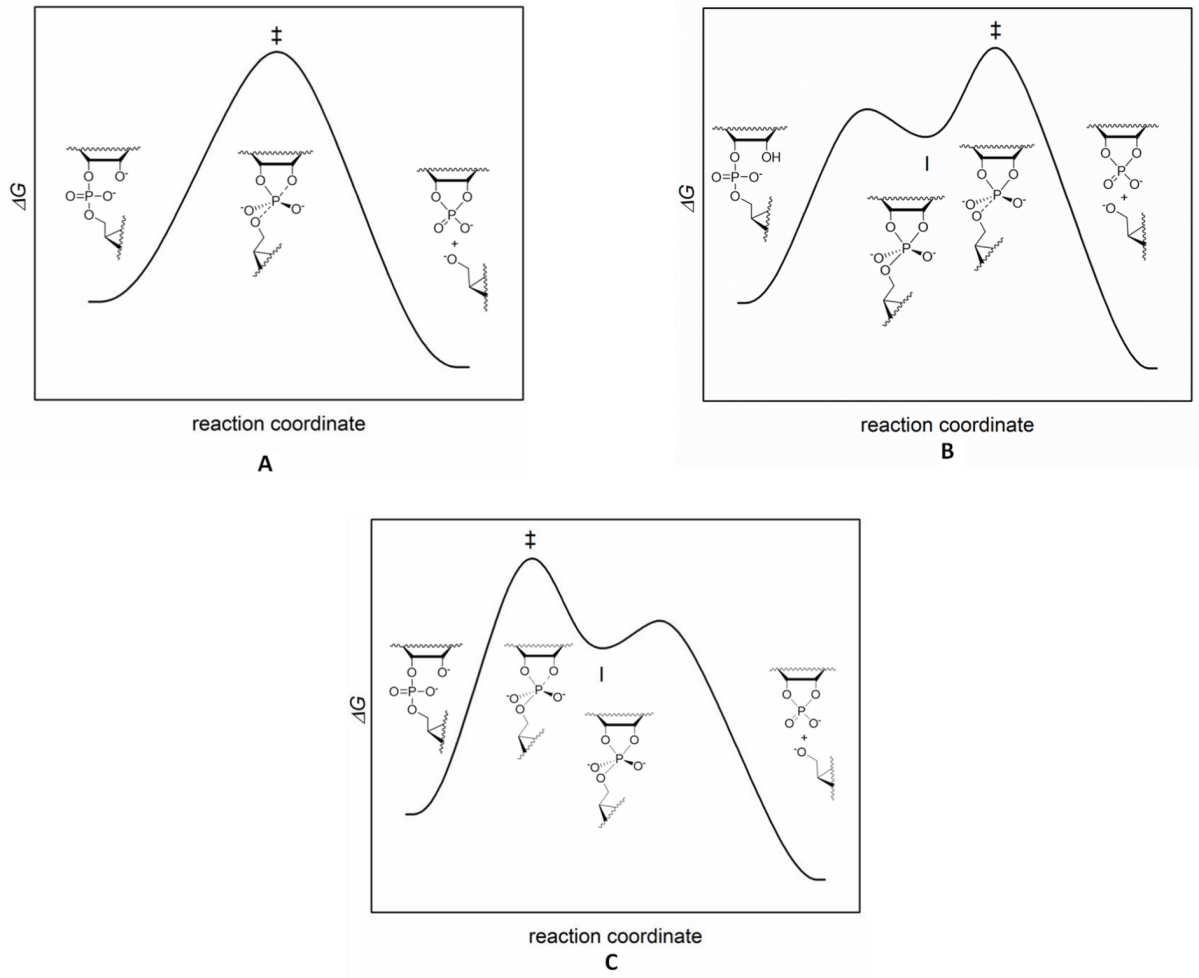
Energy profiles for a concerted A_N_D_N_ (A) and stepwise mechanisms (A_N_ + D_N_) with rate-limiting breakdown (B) and rate-limiting formation (C) of intermediate **I** that has a finite life-time. Hydroxide-ion-catalyzed cleavage of RNA has been used to exemplify alternative mechanisms. In reality, the reaction takes place by rate-limiting breakdown of the intermediate (B).

Non-enzymatic cleavage of phosphodiester linkages of nucleic acids proceeds by an intra- (RNA) or intermolecular (DNA) nucleophilic attack on phosphorus. The reaction proceeds via a pentacoordinated species having the structure of a trigonal bipyramid. In case this species represents an energy maximum on a single barrier energy profile, as with S_N_2 displacement at carbon, the reaction is called concerted and the pentacoordinated species is a transition state. The reaction is a synchronous displacement (A_N_D_N_) when bond formation to the entering nucleophile is as advanced as bond fission to the departing nucleophile (A in Figure 2). In case the bond formation is more or less advanced than the bond fission, the reaction still is concerted but has an associative or dissociative nature, respectively. The pentacoordinated species, called pentaoxyphosphorane, may also have a sufficiently long life-time to represent a minimum on the energy profile. The reaction then proceeds in a stepwise manner. It is an associative nucleophilic displacement (A_N_ + D_N_) with late transition state if the barrier for breakdown of the phosphorane intermediate to products is higher than the barrier for formation of the intermediate (B in Figure 2). If the barrier for the phosphorane formation is higher than the barrier for its breakdown to products, the transition state is early and formation of the phosphorane is rate-limiting (C in Figure 2). The phosphorane intermediate may still have a finite life-time, but experimental distinguishing between this kind of a reaction and a concerted displacement is difficult.

**Figure 3:**
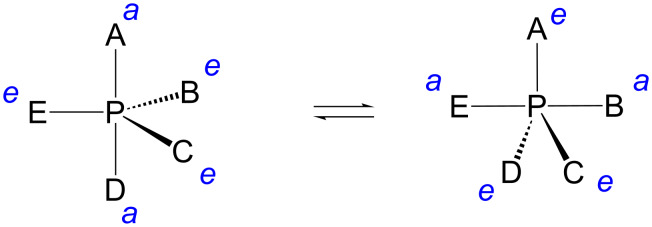
Pseudorotation of a trigonal bipyramidal phosphorane intermediate by Berry pseudorotation [20].

Two of the ligands within the bipyrimidal phosphorane take an apical (*a* in Figure 3) and the rest an equatorial (*e* in Figure 3) position. According to the so-called Westheimer´s rules [19], nucleophiles enter and depart the phosphorane intermediate only through an apical position. Electronegative ligands prefer an apical position, while negatively charged oxygens are locked to an equatorial position. Bulky ligands tend to be equatorial. If two of the oxygen atoms are bridged by an ethylene group, as in the phosphorane obtained by the attack of 2´-OH of RNA on phosphorus, one must be apical and the other equatorial. A sufficiently stable phosphorane may, however, undergo a structural change known as Berry pseudorotation [20]: one of the equatorial ligands remains equatorial, while the rest turn apical and the apical ligands equatorial. Several alternative models for isomerization of trigonal-bipyramidal pentacoordinate compounds have been presented [21], but Berry pseudorotation has almost exclusively used in mechanistic discussion of RNA cleavage.

The stability of the phosphorane intermediate largely depends on its state of protonation. The first p*K*_a_ value of the acyclic tetraalkoxy monohydroxy phosphorane has been estimated to be 8.6 for an equatorial hydroxy group and 13.5 for an apical group [22]. For a cyclic phosphorane derived from ethylene phosphate, the first p*K*_a_ value is 7.9 and the second 14.3, both values referring to an equatorial hydroxy ligand [23]. Accordingly, both neutral phosphorane and its monoanion are present in significant amount at physiological pH. In case a dianionic phosphorane is formed, its protonation to a monoanion expectedly is thermodynamically favored, but it is not clear whether the life-time is long enough to allow this.

**Figure 4:**
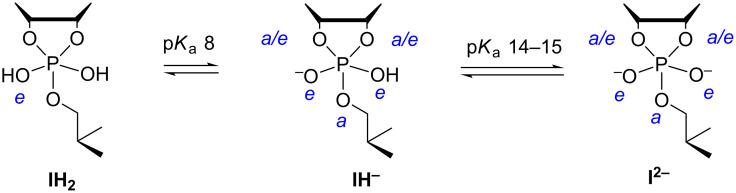
Protolytic equilibria of phosphorane intermediate of RNA transesterification.

The cyclic phosphorane intermediate of RNA cleavage is in neutral form (**IH_2_** in Figure 4) sufficiently stable to pseudorotate [24]. According to DFT calculations, the barrier for preudorotation is 10 kcal mol^−1^ lower than the barriers for breakdown of the intermediate [25]. The calculations also suggest the monoanionic form (**IH^−^**) to be able to pseudorotate, even more rapidly than the neutral form [26]. The breakdown of the phosphorane is, however, also faster than with neutral phosphorane and, hence, the life-time of the monoanion is shorter. The dianionic phosphorane (**I^2−^**) is very unstable and cannot pseudorotate, owing to the high barrier for transfer of negatively charged oxygen from equatorial to apical position. Recent DFT calculations suggest the barrier to be about 30 kcal mol^−1^ [27].

While several lines of evidence suggest that the cleavage of the RNA phosphodiester bonds proceeds via a phosphorane intermediate rather than a phosphorane-like transition state [28–30], this is not necessarily the case with DNA that is cleaved by an attack of an external nucleophile. Recent hybrid quantum mechanical/effective fragment potential (QM/EEP) calculations on the hydroxide-ion-catalyzed hydrolysis of diethyl phosphate monoanion, however, suggest that the acyclic phosphorane obtained still is an intermediate [31]. The lifetime for the dianionic pentacoordinated species obtained by the attack of the hydroxide ion on the phosphorus has been argued to represent an energy minimum between the transition states for the attack of HO^−^ and the departure of EtO^−^ and to have a lifetime of 1 picosecond. With leaving groups that are less basic than EtO^−^, such as 5´-O^−^ of nucleoside, the lifetime expectedly is shorter. If the leaving group is very good, such as an aryl group, a synchronous concerted mechanism (A_N_D_N_) may take over the stepwise mechanism (A_N_ + D_N_).

### Model compounds and experimental tools

Studies with phosphodiester models are aimed at providing firm mechanistic understanding of the hydrolysis and transesterification reactions of nucleic acids. Such information is indispensable for critical evaluation of mechanistic proposals of more complicated enzymatic processes and for the development of artificial cleaving agents that have enzyme-like catalytic properties but are more robust. pH-Rate profiles, linear free energy relationships and kinetic heavy atom isotope effects are the experimental approaches that are, together with construction of multifunctional cleaving agents, most extensively used in mechanistic studies of small molecular phosphodiester models. Kinetic studies over a wide pH-range allow division of observed rate constants to contributions of different ionic forms and, hence, the upper limit for the effect of protonation or deprotonation of a particular atom on the rate is obtained [29,32]. Linear free energy relationships are, in turn, used to determine the position of transition state on the reaction coordinate [33]. The polar property of either entering or departing nucleophile or non-departing groups is altered in a systematic manner and the effect on reaction rate is compared to the effect on the equilibrium of the reaction. In this manner, information about charge distribution in the transition state is obtained; whether the transition state is early (close to starting materials) or late (close to products). A free energy relationship is in principle a plot of activation free energy, Δ*G*^‡^ (or log *k*), against the change in standard free energy of the reaction, Δ*G*^o^ (or log *K*_eq_). The latter quantity is often difficult, sometimes even impossible, to determine. For this reason, Δ*G*^‡^ (or log *k*) is more frequently plotted as a function of the p*K*_a_ of the departing (or entering) nucleophile. The slope of the plot, known as a β_lg_ (or β_nuc_), may have values greater than unity. It does not directly tell the position of transition state on the reaction coordinate. This parameter, the so-called Leffler´s α, is, however, obtained as a ratio of β_lg_/β_eq_ or β_nuc_/β_eq_, if a reasonably reliable estimate for the β value of the equilibrium reaction, β_eq_, is available. As long as cleavage of phosphodiesters is concerned, β_eq_ = 1.74 reported for the phosphoryl transfer of phosphono monoanion is usually used as the reference value for the equilibrium reaction [34]. Likewise, the occurrence of the proton transfer as part of the rate limiting step may be evaluated by altering the acidity of the proton donor (or acceptor). Plotting of log *k* against the p*K*_a_ of the proton donor (or acceptor) gives the Brönsted α (β for the acceptor) that refers to the extent of proton transfer in the transition state.

The kinetic heavy atom isotope effect (KIE) is a most useful tool for mechanistic studies, especially since it may be used as well in enzymatic and non-enzymatic reactions [35–36]. Replacing a single atom in the substrate with its heavy isotope has so small influence on structure that enzyme–substrate interaction is not distorted, which is the case with other structural modifications. Kinetic isotope effect is defined as the ratio of the rate constants obtained with the light and heavy isotope containing compound, KIE = ^light^*k*/^heavy^*k*. When this ratio is greater than unity, the isotope effect is called normal, otherwise inverse. KIE refers to the difference in bonding that takes place on going from ground state to transition state. The effect is a primary KIE when the isotopically labelled atom is directly involved in bond making or bond breaking in the rate-limiting step. In case the isotopic substitution occurs further in the molecule, the KIE is secondary. The primary KIE is usually normal (>1), while the secondary can be either normal or inverse. The reason is that KIE consists of two contributions, a temperature independent (TIF) and temperature dependent (TDF) factor [37]. As regards the primary KIEs, the motion along the reaction coordinate is the predominant source of KIE. The KIE for this process is normal and largely dominated by TIF. With secondary KIEs, motion along the reaction coordinate is less important and changes in TDF-dependent vibrational modes of the transition state start to play a role. That is why both normal and inverse effects are possible.

The kinetic solvent isotope effect (KSIE) is another mechanistic tool frequently used to distinguish between alternative mechanisms. KSIE is an indication of a kinetically significant proton transfer that takes place on going from initial to transition state and shows up as reactivity difference in experiments made in H_2_O and D_2_O solutions of equal pL (L = H or D). The proton transfer may, however, take place either in pre-equilibrium or rate-limiting stage. Distinguishing between these alternatines is possible, if the equilibrium isotope effect for the pre-equilibrium may be reliably estimated. In case no KSIE is observed, no proton transfer takes place in the rate-limiting step. Proton inventory studies are used to examine how many protons are transferred in the rate-limiting step. In this technique, rate constants are determined as a function of isotopic ratio *n*, and the shape of a plot *k*_n_/*k*_o_ vs *n* gives information on the proton transfer processes. Unfortunately, interpretation of the data is not always straightforward, owing to possible contribution of the equilibrium isotope effect that refers to binding of the catalyst to the phosphate group [27,38].

**Figure 5:**
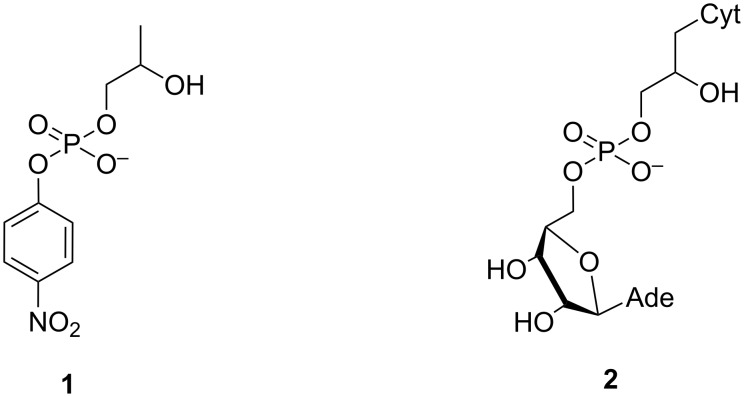
Structures of acyclic analogs of ribonucleosides.

Dinucleoside-3´,5´-monophosphates are obvious small molecular models with which to study the cleavage of phosphodiester linkages in nucleic acids. Kinetic studies with these compounds are, however, somewhat laborious, since HPLC chromatography has to be used to analyze the content of samples withdrawn at suitable intervals. That is why many research groups prefer to use a simpler model, 2-hydroxypropyl *p*-nitrophenyl phosphate (HPNP; **1**, Figure 5), the hydrolysis of which can be followed by UV-spectrophotometry. A lot of useful observations have been done with this simple model. One should, however, bear in mind that the *p-*nitrophenoxy group is a 10^8^ times better leaving group than a 5´-linked nucleoside and, hence, the rate limiting step of these two reactions can well be different, as discussed later in more detail below. In addition, the acyclic structure only poorly mimics the ribofuranosyl structure of the 3´-linked nucleoside. The acyclic analog **2**, for example, is cleaved under basic conditions 500 times less readily than a normal diribonucleoside-3´,5´-monophosphate [39]. A small molecular catalyst may accelerate the cleavage of **1** by stabilizing a rotamer that favors intramolecular attack of the neighboring hydroxy function on phosphorus, while this kind of acceleration evidently plays a minor role, if any, with ribonucleoside 3´-phosphodiesters. Finally, phosphate migration in **1** takes place between a primary and secondary hydroxy group, whereas with ribonucleoside 3´-phosphodiesters both hydroxy functions are secondary. Accordingly, extrapolation of the results obtained with **1** to the cleavage of nucleic acids is not straightforward. Care should be exercised to avoid misinterpretations.

Oligonucleotides containing a thiosubstituted nucleotide are extensively used in mechanistic studies of protein nucleases and ribozymes. Rate accelerating 3´-bridging substitution has been used to find out whether the chemical step really is rate-liming and 5´-substitution to verify that some small ribozymes utilize general acid catalysis [40]. The underlying idea behind the latter application is that protonation of the leaving group by a general acid is not needed with 5´-thiosubstituted analogs, since the sulfide ion is a much better leaving group than the alkoxide ion. Most extensively used thiosubstitution, however, is replacement of either one of the non-bridging oxygens with sulfur, which allows stereochemical studies based on the so-called rescue effect [41–42]. When non-bridging oxygen that participates in binding of Mg^2+^ is replaced with sulfur, the activity drops, but may be restored by using a soft Lewis acid, such as Mn^2+^ or Zn^2+^. The necessary background information for the studies with thiosubstituted oligonucleotides has been obtained by comparative studies with similar analogs of dinucleoside-3´,5´-monophosphates [43].

### Cleavage of RNA by Brönsted acids and bases

#### Buffer-independent reactions

**Figure 6:**
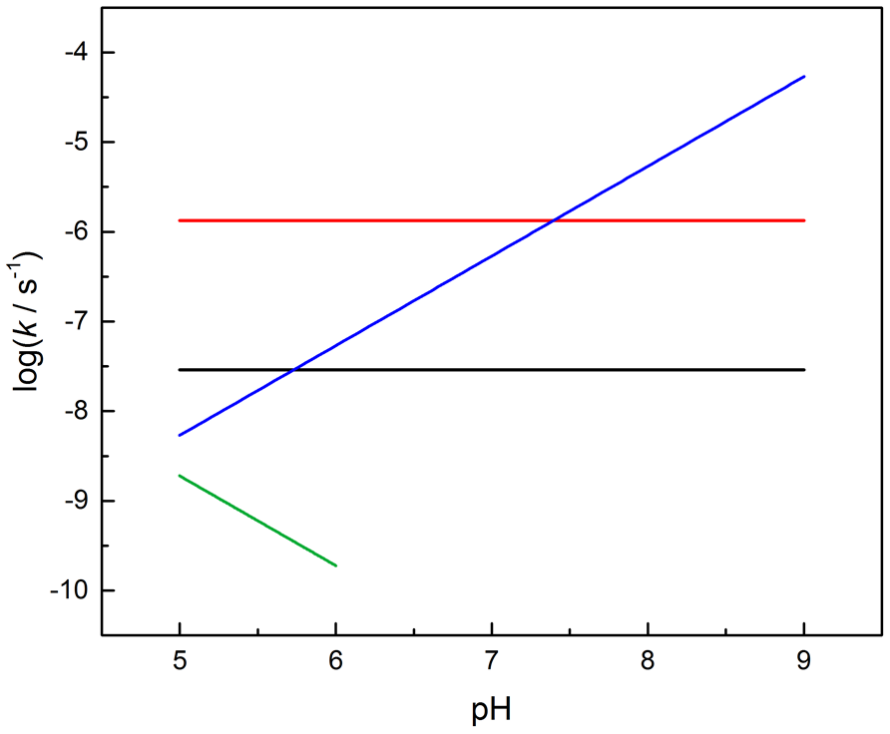
First-order rate constants for buffer-independent partial reactions of uridyl-3´,5´-uridine at pH 5–9 and 90 °C. Hydronium-ion-catalyzed isomerization (green), hydroxide-ion-catalyzed cleavage (blue), pH-independent cleavage (black), pH-independent isomerization (red). Based on the data from ref*.* [44].

**Scheme 1:**
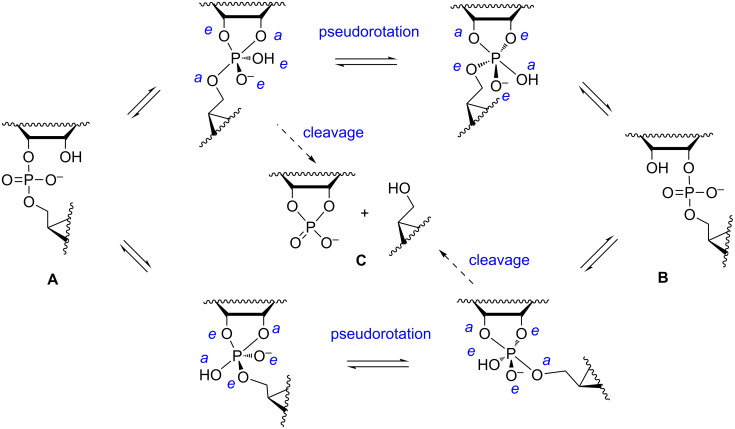
pH- and buffer-independent cleavage and isomerization of RNA phosphodiester linkages. Observed first-order rate constant for the cleavage (*k*_cl_) refers to transesterification of **A** + **B** to **C**, and observed rate constant for isomerization (*k*_is_) to mutual isomerization of **A** and **B**, the values for the forward and reverse reactions being almost equal.

The predominant buffer-independent reactions of RNA phosphodiester linkages at physiological pH (pH 6–8) are pH-independent isomerization to 2´,5´-bonds (red line in Figure 6) and hydroxide-ion-catalyzed transesterification to a 2´,3´-cyclic phosphate by departure of the 5´-linked nucleoside, followed by subsequent hydrolysis to a mixture of 2´- and 3´-phosphates (blue line in Figure 6) [44–45]. These reactions are approximately as fast at pH 7, the isomerization being faster under more acidic and cleavage under more basic conditions. The occurrence of isomerization inevitably shows that the monoanionic phosphorane, most likely obtained by the attack of 2´-OH on the phosphorus atom with concomitant transfer of the proton to the non-bridging oxygen [46–47], is able to pseudorotate at physiological pH. It is not quite clear whether the pseudorotation takes place through the monoanionic species or kinetically invisible protonation to more stable neutral phosphorane. DFT calculations suggest that the monoanionic form really is stable enough to pseudorotate and the breakdown of the intermediate to 2´- or 3´-phosphodiesters is approximately as fast as the pseudorotation [25]. According to the same calculations, the exocyclic fission of the intermediate to a 2´,3´-cyclic phosphate, leading to pH-independent cleavage, is much slower (Scheme 1). The rate of this reaction (black line in Figure 6) is only 2% of the interconversion rate of 2´,5´- and 3´,5´-diesters [44]. Studies with various uridine 3´-alkylphosphates have, however, verified the existence of this reaction [48].

**Scheme 2:**
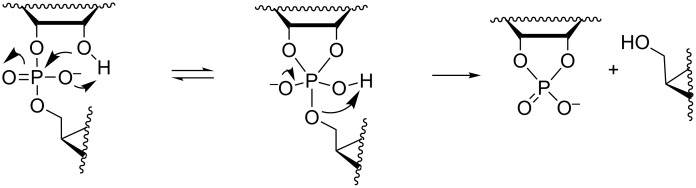
Mechanism for the pH- and buffer-independent cleavage of RNA phosphodiester linkages.

The mechanism of the pH-independent cleavage reaction has been elucidated by comparative studies of β_lg_ values. While the isomerization rate is almost independent of the polar nature of the esterified alcohol, the cleavage rate is markedly increased with the increasing electronegativity of the alkyl group. For example, the ratio of *k*_cl_/*k*_is_ is 0.014 and 1.8 with the ethyl and 2,2,2-trichloroethyl esters, respectively [48]. The β_lg_ = −0.59 is more negative than the β_lg_ = −0.12 of the acid-catalyzed cleavage, proceeding by departure of neutral alcohol, but less negative than the β_lg_ = −1.28 of the hydroxide-ion-catalyzed reaction where the departing group is an alkoxide ion [49]. Accordingly, the departing oxygen atom seems to become protonated concerted with rate-limiting rupture of the P–OR bond. The essential mechanistic features, hence, are proton transfer to non-bridging oxygen concerted with the attack of 2´-OH, which increases the nucleophilicity of O2´ and stabilizes the phosphorane intermediate, and proton transfer from the non-bridging oxygen to the departing oxygen, which destabilizes the phosphorane and stabilizes the leaving group (Scheme 2). Combined QM/MM simulations have lent support for this interpretation [47]. With triester analogs, such as uridine 3´-diethyl phosphate, the latter intramolecular proton transfer is not possible and the ratio *k*_cl_/*k*_is_ is much smaller than with the diester analog, around 10^−5^ [50]. Since the barrier for the endocyclic cleavage of the phosphorane intermediate is more than 10 kcal mol^−1^ lower than that for the exocyclic cleavage, it is not clear whether a similar proton transfer from a phosphorane hydroxy ligand to the departing oxygen occurs concerted with the fission of P–O2´ and P–O3´ bonds or does protonation of these oxygens take place after the bond fission.

**Scheme 3:**
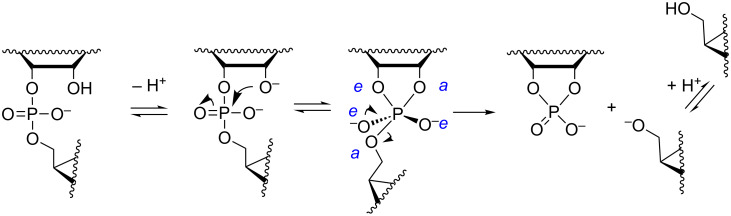
Hydroxide-ion-catalyzed cleavage of RNA phosphodiester linkages.

The hydroxide-ion-catalyzed cleavage that dominates at pH >7.5, proceeds by pre-equilibrium deprotonation of the 2´-OH and subsequent attack of the 2´-oxyanion on the phosphorus atom of a monoanionic phosphodiester linkage, giving a dianionic phosphorane that decomposes to 2´,3´-cyclic phosphate by departure of the 5´-linked nucleoside as an alkoxide ion (Scheme 3). The stability of the dianionic phosphorane has been studied by experimental and computational methods. As mentioned above, the β_lg_ value of the reaction of uridine 3´-alkyl phosphates is very negative, −1.28, suggesting that the cleavage of the P–O5´ bond is rather advanced in the transition state. However, the β_lg_ value obtained with uridine 3´-aryl phosphates is much less negative, −0.54 [51]. When the data of alkyl and aryl esters is included in the same free energy plot, a break at p*K*_a_ of 12.4 occurs, i.e., close to the p*K*_a_ of the attacking 2´-OH [52]. A free energy plot exhibiting a breakpoint at the p*K*_a_ of the attacking nucleophile is usually taken as a rather compelling evidence of a change in the rate-limiting step [33], in this case from the formation of the phosphorane intermediate with aryl esters to breakdown of this intermediate with alkyl esters. The results of DFT calculations lend further support to this interpretation and suggest that the 2,2,2-trichloroethoxy group is an example of an alkyl leaving group where the barrier for the formation of phosphorane intermediate still is slightly higher than the barrier for its departure [15].

Assuming that the β_eq_ = −1.7 reported for the phosphoryl transfer of phosphono monoanion [34] is valid for the hydroxide-ion-catalyzed cleavage of RNA phosphodiester bonds, the highly negative β_lg_ value, −1,28, means that Leffler´s α referring to the fraction of total bond cleavage is 0.7. The β_nuc_ value, in turn, helps to evaluate how advanced the formation of the P−O2´ bond is. This parameter has been determined by incorporating 2´-*C*-X-uridines (X = H, Me, CFH_2_, CF_2_H, CF_3_) into an oligodeoxyribonucleotide and plotting the cleavage rate against the p*K*_a_ of the 2´-OH [53]. The value obtained, β_nuc_ = 0.75, means that the P–O2´ bond is approximately half formed (Leffler´s α ≈ 0.4–0.5) in the transition state.

The isotope effects determined for the cleavage of 3´,5´-UpG at pH 14, i.e., under conditions where the attacking 2´-OH is almost completely deprotonated, lend further support for the mechanism in Scheme 3 [54–56]. No solvent D_2_O isotope effect occurs, consistent with rapid pre-equilibrium deprotonation of the attacking 2´-OH. For the departing 5´-O, the ^18^O KIE is normal, ^16^*k*_lg_/^18^*k*_lg_ = 1.034 ± 0.004, and for the attacking 2´-O^−^, the KIE is inverted, ^16^*k*_nuc_/^18^*k*_nuc_ = 0.984 ± 0.004 [54]. Both effects are large and consistent with advanced P–O5´ fission and P–O2´ formation in the transition state. For comparison, with uridine 3´-(*p*-nitrophenyl phosphate), the leaving group KIE expectedly is small, ^16^*k*_lg_/^18^*k*_lg_ = 1.0059 ± 0.0004, indicating that the departure of the aryloxy group is not markedly advanced [57]. The secondary KIE for the replacement of the non-bridging oxygen of the attacked phosphate is almost negligible, ^16^*k*_O1P_/^18^*k*_O1P_ = 0.999 ± 0.001 [16].

#### Buffer-catalyzed reactions

**Scheme 4:**
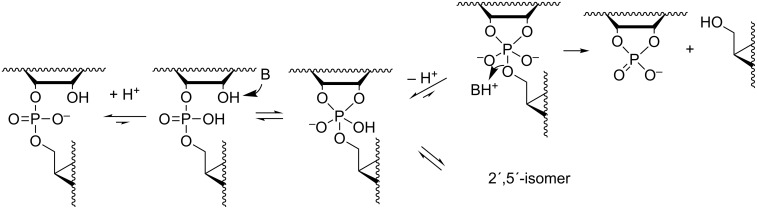
Anslyn's and Breslow's mechanism for the buffer-catalyzed cleavage and isomerization of RNA phosphodiester linkages [59].

**Scheme 5:**
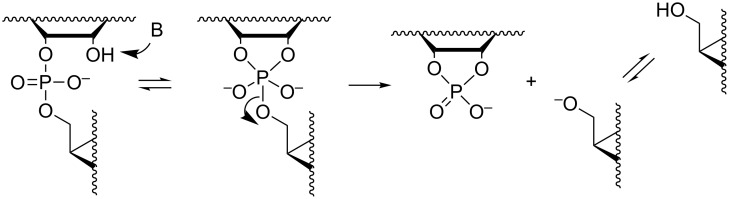
General base-catalyzed cleavage of RNA phosphodiester bonds.

While the mechanisms of buffer-independent reactions prevailing at physiological pH are rather well established, the buffer-catalyzed reactions still appear to be open to various mechanistic interpretations. The main reason for this is experimental difficulty. The buffer-dependent rate is rather modest compared to the buffer-independent rate. High buffer concentration has to be used and this makes elimination of salt and co-solute effects difficult. Since histidine residues are known to play a central role in the catalytic center of RNase A [58], one of the most extensively studied protein nucleases, catalysis by imidazole/imidazolium ion (Im/ImH^+^) buffers has been of special interest. The pioneering studies were carried out by the group of Breslow [59]. Their mechanistic suggestion is depicted in Scheme 4. Im is argued to catalyze the attack of 2´-OH on phosphorus by serving as a general base, but only if the phosphodiester linkage has undergone rapid initial protonation. In other words, a monoanionic phosphorane is obtained by a specific acid/general base mechanism that is experimentally equivalent to general acid catalysis. The monoanionic phosphorane is stable enough to pseudorotate and may, hence, undergo isomerization to the 2´,5´-diester without additional catalysis. The cleavage reaction is, in turn, suggested to take place by pre-equilibrium deprotonation of the phosphorane intermediate, followed by general acid-catalyzed fission of the P–O5´ bond; experimentally a general base catalysis is observed. An interesting feature of the mechanism is that both the formation and breakdown of the phosphorane intermediate proceed through a minor ionic form in a pre-equilibrium mixture. The mole fraction of neutral phosphodiester, for example, is in imidazole buffers of the order of 10^−6^ (p*K*_a_ of phosphodiester ≈ 1). This means that protonation of the phosphodiester linkage must facilitate the nucleophilic attack on phosphorus by at least a factor of 10^6^. As regards deprotonation of monoanionic phosphorane, the p*K*_a_ is around 14 [23], which means that deprotonation should accelerate the general acid-catalyzed departure of the 5´-linked nucleoside by a factor of 10^7^. The mechanistic proposal has partly been based on Breslow’s studies on hydrolysis of 4-*tert*-butylcatechol cyclic phosphate by regioisomers of β-cyclodextrins bearing two imidazole groups [60]. This reverse reaction of the cyclization of 4-*tert*-butylcatechol 2-*O*-monophosphate has been shown to proceed via a monoanionic (monoprotonated) phosphorane and, hence, argued to lend support for the mechanism in Scheme 4. This mechanism has been criticized [61–63], but also defended by a reinvestigation [64]. According to the additional studies, the original mechanistic suggestion is in principle valid, but has to be supplemented with a general base-catalyzed reaction through a dianionic phosphorane transition state (Scheme 5) that takes place in parallel with the stepwise reaction through a phosphorane monoanion (Scheme 4).

**Scheme 6:**
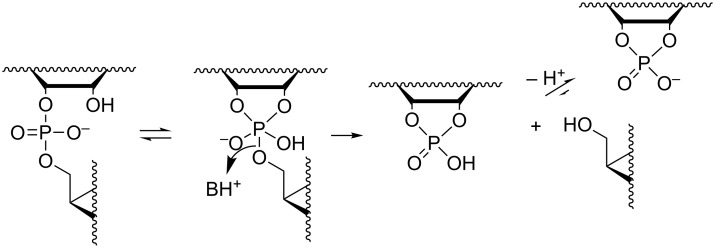
Kirby´s mechanism for the buffer-catalyzed cleavage of RNA phosphodiester bonds [65].

The group of Kirby has suggested a somewhat simpler mechanism based on two concurrent reactions: rapid initial formation of a monoanionic phosphorane that undergoes rate-limiting general acid-catalyzed cleavage (Scheme 6) and the general base-catalyzed reaction through a dianionic phosphorane transition state [65].

To avoid the contribution of buffer-independent catalysis by hydroxide ions, the buffer-catalyzed cleavage of RNA models has been studied in 80% aq DMSO (v/v). The autoprotolysis constant of water is suppressed by four orders of magnitude (p*K*_w_ = 18.38) on going from water to this mixture [66], whereas the p*K*_a_ values of amines experience only a modest change [67]. Accordingly, general acid/base catalysis may be studied with amine buffers at much lower hydroxide ion concentrations than in water. This technique was first applied by the group of Yatsimirsky to cleavage of a HPNP [38]. In 0.1 mol L^−1^ piperidine buffer, for example, the buffer-catalyzed reaction was 10^3^-fold faster than the buffer-independent reaction. The observed rate constant showed both first- and second-order dependence on the buffer concentration, *k*_obs_ = *k*_1_[B] + *k*_2_[B][BH^+^]. The Brönsted β value for the first-order term was 0.77 and this reaction was suggested to be a general base-catalyzed formation of dianionic phosphorane (Scheme 5). The second-order term, which was important especially in guanidine and amidine buffer, was assumed to refer to binding of BH^+^ to the anionic phosphodiester linkage more or less concerted with the general base-catalyzed attack of the 2´-OH. The situation seems, however, to be rather different with dinucleoside-3´,5´-monophosphates. The buffer-catalyzed reaction of UpU is not so much faster than the buffer-independent reaction, in 0.1 mol L^−1^ piperidine buffer only 4-fold faster [68]. No second-order dependence of rate on buffer concentration was observed. It should be, however, noted that kinetic measurements in the most interesting guanidine and amidine buffers failed, evidently owing to partial decomposition of the buffer constituents during the prolonged incubation at 90 °C. Both cleavage and isomerization were observed, but only the cleavage was subject to buffer catalysis, viz. general base catalysis. In aqueous solution, second-order dependence of rate on buffer concentration has never been reported.

Besides imidazole, guanidine and primary amines have received special interest as cleaving agents of RNA [69]. Guanidine is the side-chain functionality of arginine, an active component of the catalytic center of some nucleases, e.g., Staphylococcal nuclease [70] and topoisomerase [71]. Additionally, it is a substructure of guanine base that in hammerhead [72–73] and hairpin [74] ribozymes participates in proton transfer from the attacking 2´-OH to non-bridging phosphoryl oxygen. Primary amines are, in turn, used to mimic the action of the ε-amino group of lysine. Both guanidine and primary amino groups are basic functions that at physiological pH are present as guanidinium and ammonium ions. These ions tend to reduce electron density in their vicinity, inductively through bonds and electrostatically through space, or they may serve as weak general acids. The guanidine group may additionally participate in proton shuttling through various tautomeric forms [75] and the amino group through bifurcated H-bonds.

**Figure 7:**
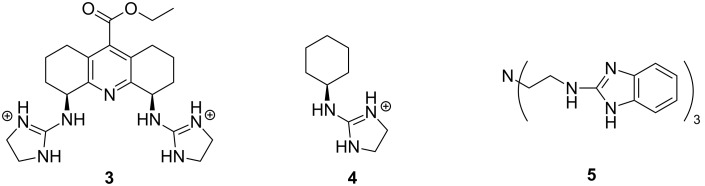
Guanidinium-group-based cleaving agents of RNA.

The first experimental observation on the ability of guanidinium containing entities to cleave RNA dates back to the early 1990s. The group of Anslyn [76] showed that compound **3** that incorporated two 2-aminoimidazolinium groups, accelerated at high micromolar concentrations the imidazole-promoted cleavage of RNA by one order of magnitude, whereas its monomeric congener **4** was ineffective (Figure 7). No detailed mechanism was suggested, but binding of **3** to the non-bridging oxygens and the departing 5´-*O* was assumed to stabilize the phosphorane intermediate and possibly protonating the departing oxygen. The second milestone on the way to guanidine-based cleaving agents was the finding that tris[2-(benzimidazol-2-ylamino)ethyl]amine (**5**) could rather rapidly degrade RNA [77]. The first-order rate constant for the cleavage of an individual phosphodiester linkage of a 30-mer RNA sequence was 3.3∙10^−6^ s^−1^ at [**5**] = 1 mmol L^−1^ and 37 °C. Aggregation of **5** with RNA prevented detailed mechanistic studies. The catalyst was, however, active even in the non-aggregated state, though possibly somewhat less efficient. The p*K*_a_ value of the 2-aminobenzimidazolium ion is about 7, being exceptionally low for a guanidinium compound. This low basicity was suggested to be a central factor behind the catalytic activity.

**Scheme 7:**
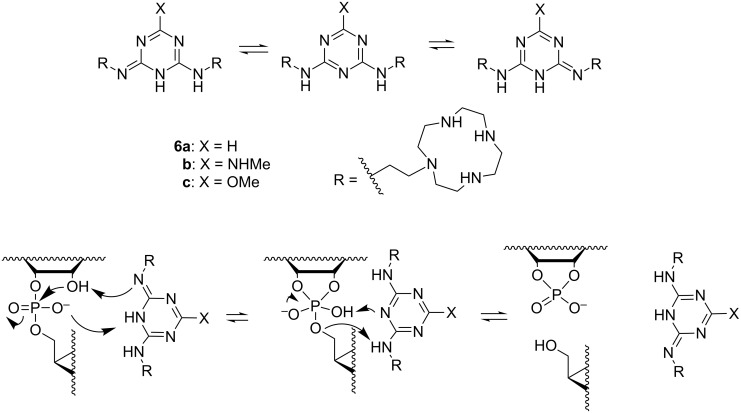
Tautomers of triazine-based cleaving agents and cleavage of RNA phosphodiester bonds by these agents [78].

A clarification of the mechanism of guanidine-based catalysis has more recently been attempted by anchoring a 2,4-diamino-1,3,5-triazine core to the N3 of uracil bases of UpU by two side arms, each bearing a Zn^2+^–cyclen complex (Scheme 7) [78]. The ternary complex of Zn^2+^, UpU and **6a** was shown to be more stable than any of the binary complexes of these species. Within this ternary complex, the triazine core could interact with the phosphodiester linkage and via various tautomeric forms facilitate the proton transfer between the attacking 2´-OH, non-bridging phosphate oxygen and departing 5´-O. The scaffold still was flexible enough to allow both cleavage and isomerization of the phosphodiester linkage. In the pH range 6–8, where the triazine core remained neutral (p*K*_a_ = 3.96), the cleavage rate was pH-independent and the acceleration at pH 7 was 30-fold compared to the buffer-independent cleavage of UpU. At pH 6, the acceleration was 100-fold. By contrast, isomerization was not accelerated. The catalytic efficiency was not sensitive to the basicity of the triazine core. More basic 6-NHMe (**6b**; p*K*_a_ = 5.28) and less basic 6-OMe (**6c**; p*K*_a_ = 3.54) substituted compounds were as efficient catalysts as their unsubstituted counterpart. Scheme 7 shows the mechanism suggested to explain the insensitivity to basicity of the general base. Increasing basicity of **6** was argued to favor the pre-equilibrium proton transfer from the 2´-OH to **4**, but at the same time **4** is weakened as a general acid that donates proton to the departing 5´-O in the rate-limiting step. The leaving group effect of the triazine-catalyzed cleavage was studied with uridine 3´-(alkyl phosphates) by using as a catalyst a truncated version of **6**, bearing only one anchoring side-arm [79]. The β_lg_ = −0.7 was of the same order of magnitude as the one, −0.59, reported for the pH- and buffer-independent cleavage, where water molecules mediate the proton shuttling.

**Figure 8:**
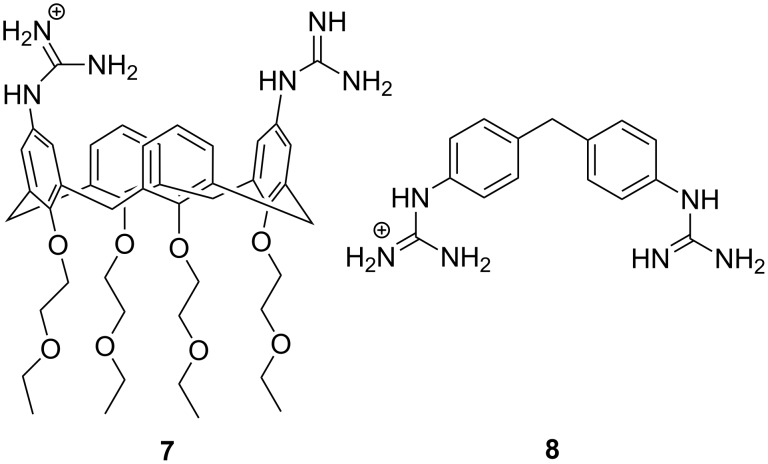
Bifunctional guanidine/guanidinium group-based cleaving agents of RNA.

**Scheme 8:**
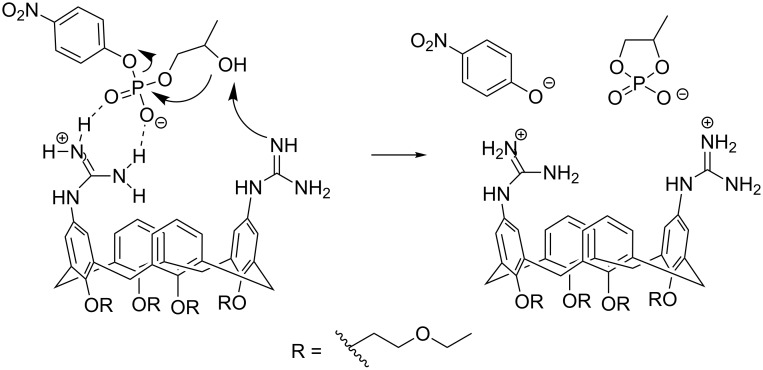
Cleavage of HPNP by 1,3-distal calix[4]arene bearing two guanidine groups [80].

Cooperative catalysis by two guanidine groups has been demonstrated by calix[4]arene derivatives **7** bearing the guanidine groups at the upper rim and *O*-(2-ethoxyethyl) groups at the lower rim [80]. The role of the latter groups was to improve solubility to hydroxylic solvents and to rigidify the calixarene system into the so-called *cone* conformation. HPNP (**1**) was used as RNA model and the reactions were carried out in 80% aq DMSO. On using a bis(guanidine)-substituted compound as a catalyst, the maximal cleavage rate was observed at pH 10.4, where only one of the two guanidines was protonated. The 1,3-distal isomer was twice as effective as its 1,2-vicinal counterpart. At 3 mmol L^−1^ concentration, the cleavage rate was 300-fold compared to the hydroxide-ion-catalyzed background reaction. It was suggested that the protonated guanidinium group binds to the phosphate group and facilitates as an electrophilic catalyst the general base-catalyzed attack of the hydroxy function on phosphorus (Scheme 8). Similar results were obtained on using diphenylmethane as a scaffold **8** (Figure 8) [81]. A cyclohexylidene or adamantylidene substituent on the methylene carbon moderately enhanced the catalytic activity. Interestingly, the calix[4]arene-based agent **7** catalyzed the cleavage of dinucleoside-3´,5´-monophosphates in 80% DMSO even more efficiently than the cleavage HPNP, the acceleration compared to the background reaction being in most favorable cases more than 10^4^-fold [78]. No saturation with the catalyst in the low millimolar range could be observed. More recent DFT calculations have led to the conclusion that replacement of the *p*-nitrophenoxide leaving group with a less electronegative nucleoside oxyanion converts the mechanism more associative, which results in more marked acceleration compared to the background reaction [27]. Dinucleoside phosphates containing uracil or guanine base were cleaved exceptionally fast [82]. No mechanistic explanation was given. Interestingly, these two bases may undergo deprotonation under mildly basic conditions (p*K*_a_ ≈ 9) in contrast to adenine and cytosine.

**Figure 9:**
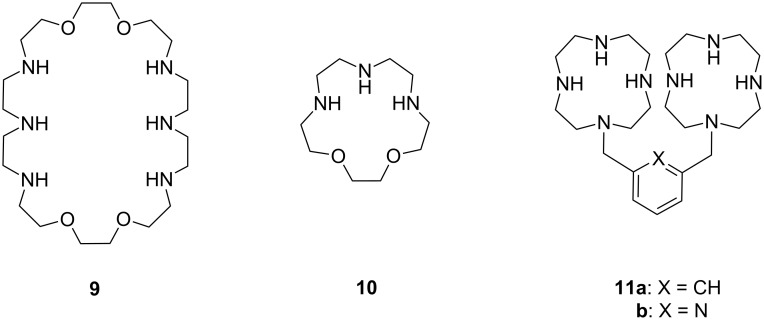
Cyclic amine-based cleaving agents of RNA.

Aliphatic amines are poor catalysts for the cleavage of RNA. The second-order rate constant for the ethylenediamine-catalyzed cleavage of ApA has been reported to be 1.2∙10^−6^ L mol^−1^ s^−1^ at pH 8 and 50 °C [83]. Cyclic polyamines are somewhat better catalysts (Figure 9). The tetracation of 1,4,16,19-tetraoxa-7,10,13,22,25,28-hexaazacyclotriacontane (**9**) cleaves ApA almost 20 times as fast as ethylenediamine, the second-order rate constant being 2∙10^−5^ L mol^−1^ s^−1^ at 50 °C [84]. The reason for this enhanced activity remains obscure. One may tentatively assume that the multiple positive charges play a role by stabilizing electrostatically the phosphorane intermediate and the departing 5´-alkoxide ion. 1,4-Dioxa-7,10,13-triazacyclopentadecane (**10**), a smaller congener of **9**, was catalytically inactive.

The tetracation of 1,3-bis(1,4,7,10-tetraazacyclododecan-1-ylmethyl)benzene (**11a**) catalyzes the cleavage, and also the isomerization, of UpU at physiological pH [85], the second-order rate constants for the cleavage and isomerization being 1.75∙10^−2^ L mol^−1^ s^−1^ and 1.5∙10^−2^ L mol^−1^ s^−1^ at 90 °C, respectively. The catalysis seems to be base moiety selective, since ApA is not cleaved. It has been suggested that one doubly charged cyclen moiety anchors the catalyst by hydrogen bonding to the carbonyl groups of uracil base and the other cyclen serves as an electrophilic catalyst by interacting with the phosphodiester linkage. The tetra- and penta-cations of 2,6-bis(1,4,7,10-tetraazacyclododecan-1-ylmethyl)pyridine (**11b**) have given similar results.

The possible role of the lysine ε-amino group in the catalytic center of RNase A has been elucidated by incorporating an amino group covalently in the vicinity of the scissile phosphodiester linkage of the model compound. For this purpose, compound **12a** bearing two aminomethyl groups at C4´ was prepared and its reactions were compared to the reactions of UpU [86] and 4´-hydoxymethyl-UpT (**12b**) [87]. The p*K*_a_ values for the mono- and diammonium ions of **12a** were determined to be 7.2 and 5.8, respectively. At pH 3–5, i.e., under conditions where both amino groups were protonated, both the cleavage and 3´,5´→2´,5´ isomerization of **12a** were pH-independent and almost two orders of magnitude faster than the corresponding reactions of UpU or **12b**. Since both reactions were accelerated, the ammonium ions were assumed to stabilize the common phosphorane intermediate, most likely by protonation of the initially formed phosphorane monoanion to a neural species. The proton transfer is thermodynamically favorable since the first p*K*_a_ value of the neutral phosphorane expectedly is around 8 [23].

**Scheme 9:**
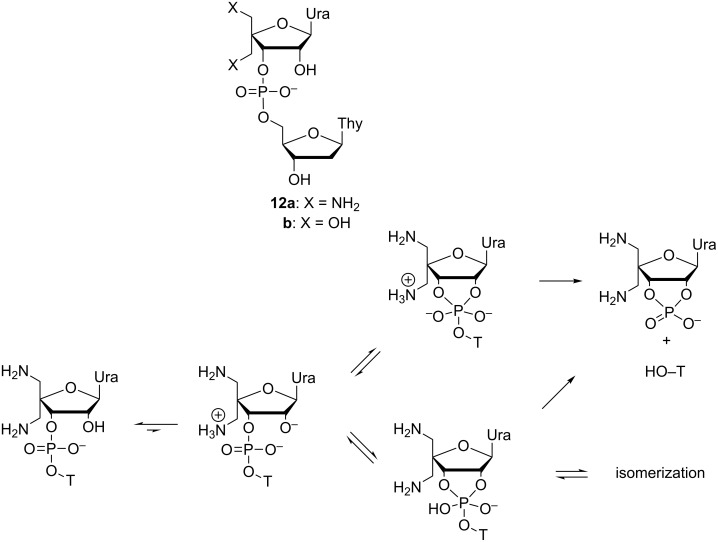
Mechanism for the pH-independent cleavage and isomerization of model compound **12a** in the pH-range 7.5–8.5 [86].

At pH > 9, the cleavage of **12a** is hydroxide-ion-catalyzed and as fast as the respective reaction of UpU and **12b**. Over a narrow pH range 7.5–8.5, where both amino groups still are deprotonated, the behavior of **12a**, however, differs from that of UpU or **12b**; another pH-independent cleavage occurs [86]. This reaction is one order of magnitude faster than the pH-independent cleavage of **12a** at pH 3–5, i.e., when both amino groups are protonated. Compared to the pH-independent cleavage of UpU, the acceleration is 10^3^-fold. It has been suggested, that the reaction proceeds through a minor tautomer having the 2´-OH deprotonated and one of the amino groups protonated, in spite of the fact that the mole fraction of this species is as low as 10^−5^. The 2’-O^−^, however, is at least a 10^6^ times better nucleophile than 2´-OH [32,88]. A dianionic phosphorane is obtained that gives the cleavage products without any kinetically visible catalysis. Concurrent with this cleavage reaction, a proton transfer from protonated aminomethyl group to non-bridging oxygen takes place more or less concerted with the PO-bond formation. A monoanionic phosphorane that is stable enough to pseudorotate is formed and, hence, isomerization takes place, although less rapidly than the cleavage (Scheme 9).

**Scheme 10:**
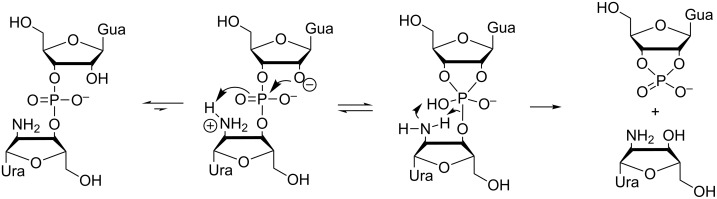
Mechanism for the pH-independent cleavage of guanylyl-3´,3´-(2´-amino-2´-deoxyuridine) at pH 6-8 [89].

Likewise, the unexpectedly fast pH-independent cleavage of guanylyl-3´,3´-(2´-amino-2´-deoxyuridine) has been accounted for by intermediary formation of a highly reactive minor tautomer (Scheme 10) [89]. The p*K*_a_ value of the amino group is surprisingly low, 4.9 at 90 °C. Both the zwitterionic (amino group protonated) and monoanionic (amino group neutral) species undergo a pH-dependent cleavage, the former at pH 3–4 and the latter at pH 6–8. Both reactions give 2´-amino-2´-deoxyuridine as the sole free nucleoside, indicating that the attacking nucleophile in both cases is the 2´-OH of the guanylyl moiety. The pH independent cleavage of the monoanion is, however, one order of magnitude faster than the cleavage of the zwitterion. This observation has led to the conclusion that the monoanion reacts through a minor tautomer having the 2´-OH deprotonated and the amino group protonated. The protonated amino group may facilitate the attack of the 2´-oxyanion by H-bonding to one of the non-bridging oxygens concerted, but upon elongation of the P–O3´ bond, the basicity of this non-bonding oxygen is decreased and the basicity of the departing O3´ is increased. Owing to this change, the H-bond to phosphate is weakened and H-bonding to O3´ is strengthened. While the reaction at pH 6–8 is 100-times faster than the cleavage of guanylyl-3´,3´-(2,5-di-*O*-methyluridine), the isomerization reaction is not accelerated by the amino substitution and, hence, only cleavage is detected at pH > 4.

### Cleavage of RNA phosphorothiolates and phosphorothioates

As discussed in the introductory part, phosphorothiolate oligonucleotides containing a bridging 3´- or 5´-thiosubstitution, are used as mechanistic probes of enzyme catalysis. Non-bridging thiosubstitution, in turn, creates *R*_P_ and *S*_P_ diastereomeric phosphorothioate linkages which have extensively been used for elucidation of the stereochemical course of enzymatic reactions and stereochemical requirements for Mg^2+^ binding. That is why, comparative kinetic studies with phosphorothioate analogs of phosphodiesters are of interest.

Bridging 3´S-substitution accelerates the hydroxide-ion-catalyzed cleavage of the phosphodiester linkage (Scheme 3) by more than two orders of magnitude, in spite of the fact that sulfur is less electronegative than oxygen and, hence, a weaker withdrawer of electrons from phosphorus [90–91]. According to theoretical calculations, the reaction is accelerated since a less strained five-membered ring is formed upon the attack of 2´-OH on phosphorus and since the polarizability of sulfur is higher than that of oxygen [16]. The heavy atom isotope effect measurements with *S*-(2-hydroxypropyl) *O*-(*m*-nitrobenzyl) phosphorothiolate have shown that the effect for the attack of the OH group, ^18^*k*_nuc_ = (1.1188 ± 0.0055), is large, suggesting an early transition state where the PO bond formation is not markedly advanced [92]. The leaving group effect, ^18^*k*_lg_ = (1.0118 ± 0.0003), is small but still present consistent with modest progress of the leaving group departure. In striking contrast to the situation with their oxygen counterparts, the 2´,3´-cyclic phosphorothiolate is clearly accumulated [90,93]. At pH 3–5, pH-independent isomerization of the 3´,5´- to 2´,5´-phosphorothiolate is faster than cleavage and 50 times as fast as the isomerization of its oxygen analog [93]. In other words, monoanionic 3´-thiophosphorane is stable enough to pseudorotate.

5´-Thiosubstitution accelerates the hydroxide-ion-catalyzed cleavage even more markedly than the 3´-substitution, the cleavage rate being from 10^4^- to 10^5^-fold compared to the oxygen analog [94–95]. With *O*-(2-hydroxypropyl) *S*-(3-nitrobenzyl) phosphorothiolate, ^18^*k*_nuc_ = 1.0245 ± 0.0047 is normal while the leaving group heavy atom KIE, ^34^*k*_lg_ = 1.0009 ± 0.0001, is very small, 1.0009 ± 0.0001, consistent with an early transition state with advanced formation of the PO bond and without appreciable lengthening of the PS bond [92]. In other words, the transition state resembles the transition of ribonucleoside 3´-aryl phosphates rather than 3´-alkyl phosphates, which is expected on the basis of 10^5^-fold lower basicity of sulfide ions compared to alkoxide ions.

The effect of non-bridging thiosubstitution on the cleavage rate is modest compared to the bridging substitutions. Phosphoromonothioates react by 100% inversion, the thioeffect, *k*_O_/*k*_S_, for the *R*_P_ and *S*_p_ isomer being 1.3 and 0.8, respectively [96–97]. Thiosubstitution tends to stabilize the dianionic phosphorane intermediate, but at the same the solvation of the phosphorane is weakened, and these two opposing influences largely cancel each other [98–100]. The solvation, hence, plays a much more important role than with 3´S*-* and 5´S-substitutions, evidently for the reason that the sulfur in non-bridging position is anionic and the charge is more dispersed than with oxygen. The leaving group effect is very similar to that with the oxygen phosphodiesters, the β_lg_ values for the alkyl and aryl esters of uridine 3´-phosphate being 1.24 [101] and 0.55 [102], respectively. This also applies to the general base-catalyzed cleavage. For the imidazole-catalyzed reaction, the β_lg_ value of uridine 3´-aryl phosphorothioates and 3´-arylphosphates are 0.63 and 0.59, respectively [102]. The thio effect, *k*_O_/*k*_S_, is somewhat greater than in specific base catalysis, ranging from 1.2 to 3.6. Altogether, the effect of non-bridging thiosubstitution on the kinetics of RNA phosphodiesters remains very modest, which makes thioates useful model compounds for the studies of rescue effect in the catalysis by large ribozymes.

Under physiological conditions, pH-independent reactions via a monoanionic phosphorane (Scheme 2) compete with the hydroxide-ion-catalyzed cleavage. At pH 5–7, these reactions even predominate [97]. Monoanionic thiophosphorane is sufficiently stable to pseudorotate, but the isomerization is moderately retarded, *k*_O_/*k*_S_, being 5 and 7 with the *R*_P_ and *S*_P_ diasteromers, respectively. The cleavage, in turn, is accelerated: *k*_O_/*k*_S_(*R*_P_) = 0.1 and *k*_O_/*k*_S_(*S*_P_) = 0.3. In addition, desulfurization takes place under these conditions. The hydrogen sulfide ion is 10^5^ times less basic than the hydroxide ion and, hence, able to compete with the sugar oxyanions as a leaving group upon breakdown of the thiophosphorane intermediate (the bond energies of P–O and P–S bonds are 86 kcal mol^−1^ and 55 kcal mol^−1^, respectively [103]). Although no desulfurization takes place at high pH, this reaction represents 80% of the disappearance of Up(s)U under neutral conditions.

Replacing both of the non-bridging oxygens in a phosphodiester linkage with sulfur does not markedly change the behavior compared to phosphoromonothioates. The thio effect, *k*_O_/*k*_S_, is 2.8 for the hydroxide-ion-catalyzed reaction, 0.2 for the pH-independent cleavage and 8 for the pH-independent isomerization [104].

### Models for the cleavage by large ribozymes

Transesterification reactions catalyzed by the large ribozymes (group I and II introns, the lariat capping ribozyme, the spliceosome and RNAse P) share a common mechanism that sets them apart from reactions catalyzed by small ribozymes or protein enzymes [42,105]. Perhaps most strikingly, the large ribozymes do not make use of the vicinal 2´-OH as a nucleophile but instead fold into an elaborate tertiary structure that allows an external nucleophile to attack the phosphorus atom of the scissile phosphodiester linkage [106–107]. The leaving group, in turn, is the 3´- rather than the 5´-oxygen. Finally, unlike many small ribozymes, large ribozymes are obligate metalloenzymes, activating the phosphodiester substrate by direct coordination of Mg(II) to the non-bridging oxygens [108–110]. All of these features present unique challenges to the design of relevant model systems.

As discussed above, non-enzymatic cleavage of RNA phosphodiester linkages proceeds exclusively by attack of the vicinal 2´-OH. No other nucleophile, including solvent water or hydroxide ion, is able to compete. The large ribozymes have to provide a solvent-free environment that suppresses the nucleophilic attack of the vicinal 2´-OH by intrachain H-bonding and promotes the attack of an external nucleophile by appropriate preorganization, or the RNA chain is locked to a conformation where intrachain in-line attack is not possible. Several approaches have been developed to simulate these conditions with small molecular models.

**Scheme 11:**
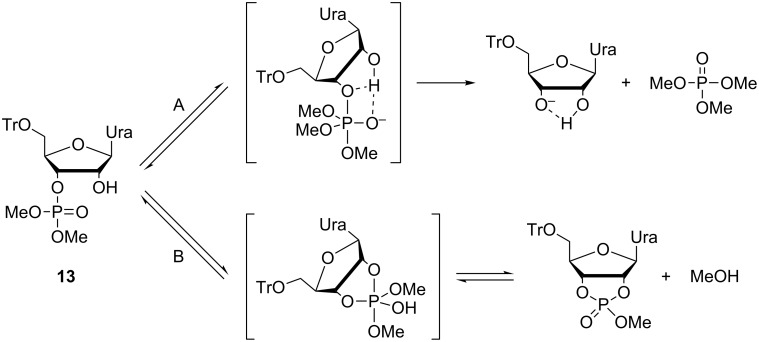
Cleavage of uridine 3´-dimethyl phosphate by A) intermolecular attack of methoxide ion and B) intramolecular attack of 2´-OH [111].

The solvent-free environment of the catalytic core of large ribozymes has been mimicked in small molecular model systems by performing the reactions in an organic solvent, rather than water. For example, intermolecular attack on a ribonucleoside 3´-phosphotriester has been observed in methanol and in a mixture of methanol and dichloromethane when methoxide ion at a high concentration was used as the nucleophile (Scheme 11) [111]. A phosphotriester, rather than a phosphodiester, was chosen as a model for better solubility in organic media as well as for higher reactivity. Regarding the overall charge, phosphotriesters can be considered to be mimics of the monoprotonated phosphodiesters.

An attack by methoxide (Scheme 11, route A) leads to release of uridine in mixtures of methanol and dichloromethane. The intramolecular attack of 2´-OH undoubtedly is much faster than the intermolecular attack of methoxide (Scheme 1, route B), but the resulting 2´,3´-cyclic triester is reverted back to the starting material by the attack of methoxide, the equilibrium in dry methanol being overwhelmingly on the side of the acyclic triester **13**. In aqueous solution, closely related triesters react exclusively by route B [88,112]. Methanolysis of the arabino and 2´-deoxyribo analogs of **13** was 30-fold slower, underlining the importance of the *cis*-diol system [111]. Apparently, the 2´-OH acts as an electrophilic catalyst which is stabilizing the negative charge developing on the phoshorane intermediate and/or the departing 3´-oxygen by H-bonding.

**Scheme 12:**
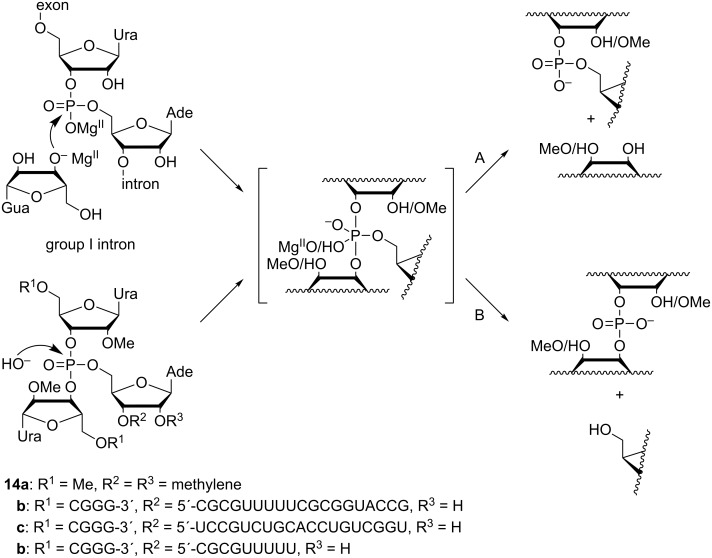
Transesterification of group I introns and hydrolysis of phosphotriester models proceed through a similar intermediate or transition state that can decompose by A) P–O3´ or B) P–O5´ bond fission.

Hydrolysis of phosphotriesters is the reverse reaction of the attack of alcohol on phosphodiesters, the key reaction catalyzed by large ribozymes. These reactions, hence, proceed through the same pentacoordinated phosphorane intermediate or transition state. Accordingly, the impact of various factors, such as intramolecular hydrogen bonding and the secondary structure around the scissile phosphate, can be studied with phosphotriester models. Hydroxide-ion-catalyzed hydrolysis of trinucleoside 3´,3´,5´-monophosphates **14a**–**d**, for example, has been used as a model reaction for transesterification of group I and II introns (Scheme 12) [113–114]. In these models, methylation of the 2´-OH group of the two 3´-linked nucleosides was necessary to prevent them from acting as intramolecular nucleophiles.

The pentacoordinated intermediate or transition state obtained by the attack of hydroxide on **14a**–**d** may decompose by cleavage of either P–O3´ (Scheme 12, route A) or P–O5´ bond (route B), yielding a 3´,5´- or a 3´,3´-phosphodiester, respectively. The ribozyme reaction follows exclusively route A [115–117], whereas hydrolysis of the model compounds (**14a**–**d**) proceeds by both routes [113–114]. With the simplest model, compound **14a**, comprising only the three nucleosides directly linked to the scissile phosphate, P–O5´ cleavage (route B) accounts for 15% of hydroxide-ion-catalyzed hydrolysis, independent of the reaction temperature (3–90 °C). The product distribution of the oligonucleotide models, **14b**–**d**, on the other hand, was temperature-dependent, the proportion of P–O5´ cleavage ranging from approximately 3% (at 3 °C) to approximately 20% (at 90 °C). Furthermore, **14b**–**d** reacted approximately 6-fold slower than **14a**. Evidently base stacking specifically retards cleavage of the P–O5´ bond. It is interesting to note that in the catalytic core of group I introns, the scissile phosphodiester linkage is embedded within a double-helical stem [118–119], where base stacking is undoubtedly stronger than in the oligonucleotide models (**14b**–**d**). Unfortunately, studying double-helical model systems was precluded by the strongly denaturing alkaline conditions required for the hydroxide-ion-catalyzed reaction to prevail.

**Scheme 13:**
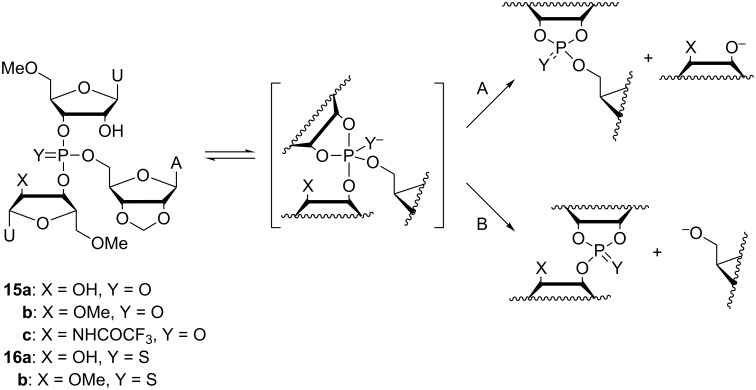
Cleavage of trinucleoside 3´,3´,5´-monophosphates by A) P–O3´ and B) P–O5´ bond fission.

Besides steric constraints of the catalytic core, stabilization of the departing 3´-oxyanion by an H-bond donated by the vicinal 2´-OH group has been proposed as an explanation for the overwhelming predominance of the P–O3´ over the P–O5´ cleavage in the reactions of large ribozymes [120–123]. Rate acceleration by a vicinal hydrogen bond donor in the leaving group has, indeed, been observed in the intramolecular cleavage of ribonucleoside 3´-phosphodiesters [89,124] as well as in the intermolecular methanolysis of ribonucleoside 3´-phosphotriesters discussed above. However, while consistent with stabilization of the leaving group, these results are open to another interpretation, viz. stabilization of the phosphorane intermediate. Hydrolytic reactions of ribonucleoside 3´-phosphotriesters featuring two different leaving groups have been studied to distinguish between these two alternatives [125–128]. Specific acceleration of departure of the leaving group with a vicinal hydrogen bond donor (Scheme 13, route A) would suggest stabilization of the leaving group, whereas equal acceleration of both of the parallel reactions (routes A and B) would be more consistent with stabilization of the common intermediate.

In both the phosphate and the phosphorothioate series, cleavage of the model triesters with a free 2´-OH group in the 3´-linked departing nucleoside **15a** and **16a** was approximately 30-fold faster than the respective reaction of the 2´-O-methylated analogues **15b** and **16b** [125–126,128]. A 2´-trifluoroacetamido group proved somewhat more activating, compound **15c** being hydrolyzed approximately 50-fold faster than **15a** [127]. In the case of **15a** and **16a**, both P–O3´ and P–O5´ cleavage (Scheme 13, routes A and B, respectively) were equally facilitated, suggesting that the 2´-OH donates a hydrogen bond to non-bridging oxygen of the phosphorane intermediate, rather than the departing 3´-oxygen. With **15c**, on the other hand, specific acceleration of P–O3´ cleavage was observed, consistent with hydrogen bond stabilization of the leaving group.

Isomerization of the internucleosidic phosphodiester linkages is not observed with ribozymes but the respective reaction of model compounds is still useful when making mechanistic interpretations, as it shares a common intermediate with cleavage. With the model triesters **15a**–**c** and **16a**,**b**, isomerization becomes hydroxide-ion-catalyzed already at pH 2 and is much faster than cleavage under neutral and alkaline conditions. Isomerization of the phosphate models was too fast to be measured but with the phosphorothioate models, comparision of the rates of hydroxide-ion-catalyzed isomerization of **16a** and **16b** was possible [128]. Interestingly, **16a** was isomerized an order of magnitude faster than **16b**, offering perhaps the most compelling piece of evidence for hydrogen bond stabilization of the phosphorane (or thiophosphorane) intermediate.

Steric constraints imposed by the tertiary structure of the large ribozymes undoubtedly have a profound effect on the course of the ribozyme-catalyzed reactions and such effects are notoriously difficult to duplicate in small molecular models. For example, the apparent discrepancy between the results obtained with simple triester models and modified ribozymes on the effect of the 2´-OH of the departing 3´-linked nucleoside could be explained in terms of an intricate hydrogen bonding network at the catalytic core of the large ribozymes [120,129–131]. On the other hand, even the simple expansion of a trinucleoside phosphotriester (such as **15b**) with short homothymine oligonucleotide arms stabilized the phosphotriester core toward hydroxide-ion-catalyzed cleavage by an order of magnitude and completely suppressed P–O5´ cleavage [132]. Even higher stabilizations were observed with more elaborate phosphate-branched oligonucleotide models [133] but the data did not allow unambiguous correlation of structure and stability. Clearly, as the model systems start to approach the large ribozymes in complexity, the results may become more relevant but at the same time more difficult to interpret.

### Cleavage of DNA by Brönsted acids and bases

The sugar-phosphate backbone of DNA is known to be extremely stable at pH 7 and 25 °C. In fact, no reliable estimate for the half-life of the cleavage of an individual 3´,5´-phosphodiester linkage is available. The estimate for the fission of a P–O bond, based on hydrolysis of dineopentyl phosphate, is 7∙10^−16^ s^−1^, corresponding to a half-life of 31 million years [2]. Most likely, the cleavage of the C5´–O bond in DNA is somewhat faster. For comparison, 99% of the hydrolysis of dimethyl phosphate proceeds under neutral conditions by nucleophilic attack on carbon leading to C–O bond cleavage [134]. Since C5´ is relatively open for a nucleophilic attack, C–O bond cleavage may take place with DNA phosphodiester linkages. In addition, depurination and various base moiety modifications may well lead to sugar ring opening that allow chain cleavage by elimination [135].

The hydrolysis of dineopentyl phosphate, taken as a model of P–O bond cleavage in DNA, is pH independent over a wide pH range from pH 7 to 12 [2], in striking contrast to cleavage of RNA which turns hydroxide-ion-catalyzed already at pH 5 [44]. Either, water attacks on the phosphorus atom of the dineopentyl phosphate monoanion, possibly by concerted proton transfer to one of the non-bridging oxygens, or hydroxide ion attacks neutral dineopentyl phosphate. In both cases the reaction takes place through a monoanionic pentacoordinated species, which may have a finite life-time. Computational calculations have provided considerable evidence for the former of these mechanistic alternatives [136].

Owing to the extremely high stability of DNA phosphodiester linkages at physiological pH, no mechanistic studies with dimeric DNA fragments have been carried out. Instead, plasmic supercoiled DNA consisting of thousands of base pairs is usually used as a target on developing various cleaving agents. Cleavage of even one phosphodiester linkage may lead to electrophoretically detectable relaxation of the supercoiled structure (Form I), first to a circular DNA (Form II) by bond cleavage within one of the chains and then to a linear form (Form III) by cleavage of both strands. Table 1 depicts structures of nonmetallic agents shown to cleave supercoiled DNA at physiological pH in aqueous solution by a hydrolytic mechanism. Cleavage by a radical mechanism has usually been excluded by showing that radical scavengers do not retard the reaction or by showing that the linearized (Form III) plasmid is a substrate of ligases. Otherwise the mechanistic information is scanty. The common feature of the cleaving agents is a dicationic structure. In addition, the agent may contain an aromatic moiety that enhances intercalation (**18**, **20**) or a hydroxy function that can serve as an intracomplex nucleophile (**20**–**22**). With the latter compounds, the guanidinium type structure has been assumed to interact with the non-bridging phosphoryl oxygens and, hence, facilitate the attack of the covalently attached hydroxy function.

**Table 1:** Cleavage of supercoiled DNA by nonmetallic cleaving agents.

compound	structure of the cleaving agent	efficiency of cleavage	ref.

**17**	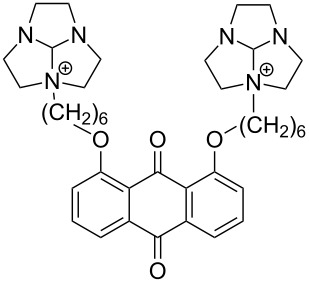	plasmid pBR322 conversion to Form III was detected upon 2 h incubation with **17** (200 mmol L^−1^) in tris buffer at pH 7.2 and 37 °C.	[137]
**18**	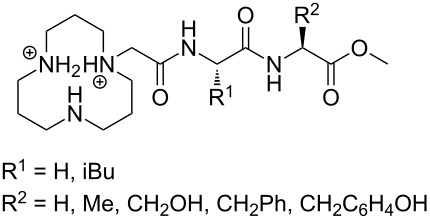	half-life for the cleavage of plasmid pUC19 to Form II reported to be 3.3 h at physiological pH.	[138]
**19**	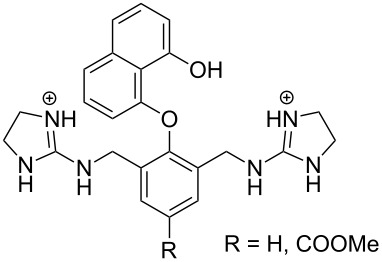	50–60% of plasmid was converted to Form II upon 48 h incubation with **19** (200 mmol L^−1^) in HEPES buffer at pH 7.2 and 37 °C.	[139]
**20**	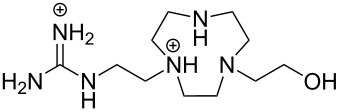	37% of plasmid pUC19 was converted to Form II upon 20 h incubation with 10 mmol L^−1^ **20** in HEPES buffer at pH 7.0 and 37 °C.	[140]
**21**	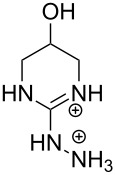	half-life for the conversion of plasmid pUC 19 to Form II 4.3 h (tris buffer pH 7.2) at saturating concentrations of **21**.	[141]
**22**	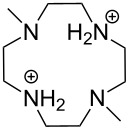	half-life for the conversion of plasmid pUC 19 to Form II reported to be 18 h (Tris buffer pH 6.0, 37 °C) at saturating concentrations of **22**.	[142]

### Metal-ion-promoted cleavage of nucleic acids

#### General

**Figure 10:**
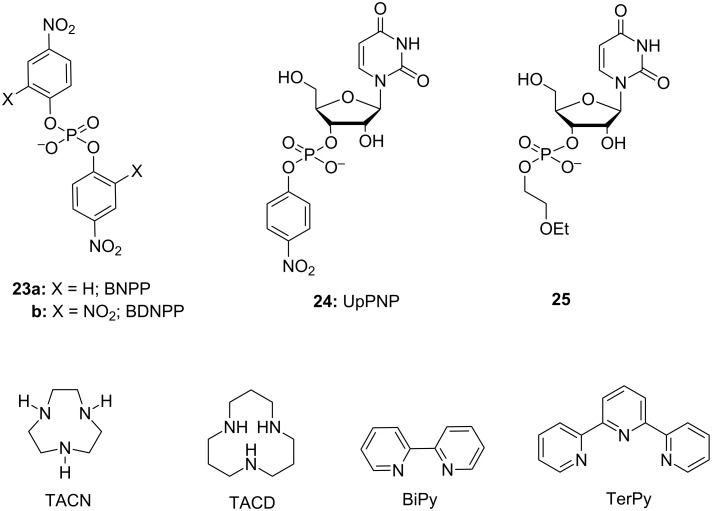
Model compounds (**23**–**25**) and metal ion binding ligands used in kinetic studies of metal-ion-promoted cleavage of nucleic acids.

Many metal ions and their complexes enhance the cleavage of phosphodiester bonds. In some cases the process is catalytic and the metal ion catalyst converts an excess of substrate into products. True catalysis with multiple turnover is generally observed with bis(*p*-nitrophenyl) phosphate (BNPP, **23a**, Figure 10) [143–144], a widely used simple model compound mimicking DNA phosphodiester bonds, and sometimes with HPNP (**1**) [145]. Usually, though, it is not the case, as the products bind to the catalyst much more strongly than the starting material. The catalyst is consumed, and the process is, strictly speaking, not catalytic. These terms are, however, used throughout the review along with more correct expressions to promote and to enhance. The rate-enhancement by metal aqua ions on the hydrolysis of DNA models and transesterification of RNA models generally is rather modest, as is shown by the chosen representative examples in Table 2. Among divalent metal ions, Zn^2+^ and Cu^2+^ are usually the most efficient ones. Alkaline and alkaline earth metal cations show only a slight rate-enhancement, whereas trivalent lanthanide ions are generally more efficient catalysts than divalent metal ions [146–148].

**Table 2:** Catalytic activity (*k*_rel_ = *k*_obs_/*k*_uncat_) of chosen metal ions and their complexes under given conditions ([catalyst], pH and temperature).

catalyst	*k*_rel_ BNPP (**23a**)	*k*_rel_ HPNP (**1**)	*k*_rel_ UpPNP (**24**)	*k*_rel_ NpN (UpU or **25**)^a^

Zn^2+^(aq)		150; 0.5 mmol L^−1^, pH 7.00, 37 °C^b^	33; 10 mmol L^−1^, pH 5.9, 25 °C^c^	32; UpU, 1 mmol L^−1^, pH 7.00, 80 °C^b^
Cu^2+^(aq)	27; 0.1 mmol L^−1^, pH 6.50, 75 °C^d^			
Eu^3+^(aq)		7700; 0.5 mmol L^−1^, pH 7.00, 37 °C^b^		475; UpU, 1 mmol L^−1^, pH 7.00, 80 °C^b^
Cu^2+^-TerPy	ND**^e^**	52; 2 mmol L^−1^, pH 7.0, 25 °C^f^	179; 10 mmol L^−1^, pH 6.6, 25 °C^c^	2164, UpU, 10 mmol L^−1^, pH 6.6, 90 °C^c^
Cu^2+^-BiPy	2000; 1 mmol L^−1^, pH 6.50, 75 °C^d^	144; 2 mmol L^−1^, pH 7.0, 25 °C^g^	116; 10 mmol L^−1^, pH 6.6, 25 °C^c^	291, UpU, 10 mmol L^−1^, pH 6.6, 90 °C^c^
Cu^2+^-TACN	5700; 2 mmol L^−1^, pH 7.0, 50 °C^h^	298; 2 mmol L^−1^, pH 7.0, 25 °C^h^		
Zn^2+^-TACD	10000; 10 mmol L^−1^, pH 8.5, 35 °C^i^	450; 0.20 mmol L^−1^, pH 7.0, 25 °C, 50% MeCN^j^	58; 10 mmol L^−1^, pH 5.9, 25 °C^c^	410; **25**, 2 mmol L^−1^, pH 6.6^c^

^a^The p*K*_a_ of the leaving group alcohol in **25** is the same as in dinucleoside monophosphates; ^b^from ref. [146] ; ^c^from ref. [149]; ^d^from ref. [150]; ^e^no catalysis has been observed as discussed in ref. [151]; ^f^from ref. [152]; ^g^from ref. [153]; ^h^from ref. [154]; ^i^from ref. [155]; ^j^from ref. [156].

In addition to the rather modest rate enhancement, studies with metal aqua ions are limited by precipitation of catalysts as hydroxides, in some cases even at neutral pH [157]. While in the case of divalent metal ions the formation of an insoluble hydroxide decreases catalytic activity, lanthanide aqua ions form gel-like material of unknown structure that is catalytically more active than aqua ions [148,158]. The reaction order in lanthanide and hydroxide ion concentration approaches three when reaching the pH where precipitation starts. Furthermore, the remarkably large rate enhancement is observed only when the gel is being formed during the course of the phosphoester cleavage.

The solubility problem can be, to some extent, overcome by the use of sufficiently stable metal ion complexes. The ligand affects the catalytic activity of metal ion and many Zn^2+^ and Cu^2+^ complexes are more efficient as catalysts than the corresponding aqua ions (Table 2). Zn^2+^ complexes of polyazamacrocycles such as 1,5,9-triazacyclododecane (TACD), 1,4,7-triazacyclononane (TACN), and their derivatives [159–160], as well as Cu^2+^ complexes of terpyridine (TerPy), bipyridine (BiPy) and their derivatives, are among the most frequently studied species. In the case of lanthanide ions, the situation is opposite. Complex formation decreases the observed catalytic activity, at least partly due to blocked gel formation. Furthermore, lanthanide complexes with neutral ligands tend to be unstable and ligands with side arms that encapsulate the lanthanide ions are required [161–162]. Ligands with negatively charged side arms form the most stable complexes, but a negative charge generally decreases the catalytic activity. In addition to improved solubility, a ligand may enable ligation of the metal complex to various structures. This is necessary in a number of applications, which are outside the scope of the present review.

**Figure 11:**
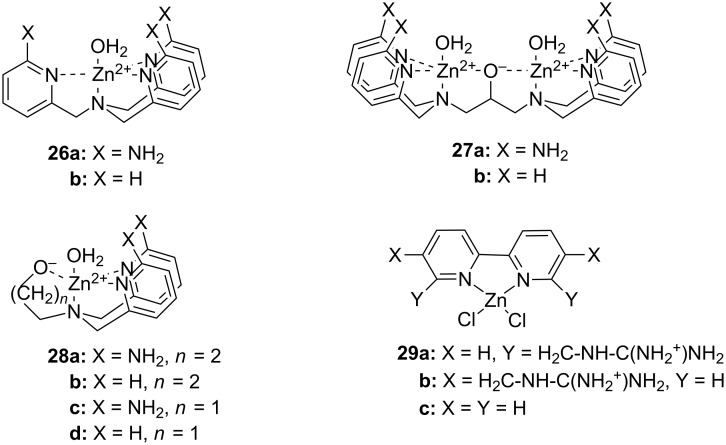
Zn^2+^-ion-based mono- and di-nuclear cleaving agents of nucleic acids.

As suggested by Breslow [163] and Chin [164] already in early 1990’s, a second metal ion [165–167] or a hydrogen bond forming substituent [168–171] can markedly enhance the catalytic activity. As an example, **26a** is a 79 times more efficient catalyst for HPNP cleavage than **26b** devoid of amino groups [168] and the rate-accelerating effect of the second metal ion center in **27b** is even more prominent when compared to **28d** [167]. A similar effect has been observed on using BNPP as a substrate: **28a** promotes the hydrolysis of BNPP 230 times as efficiently as **28b** [172] and *k*_cat_/*k*_0_ values reported for hydrolysis promoted by **29a** and **29b** are 640 and 250 times higher than that for the unsubstituted complex **29c** [173]. The higher cleaving activity partially results from stronger interactions with the substrate, but also from enhanced catalytic efficiency [173]. The importance of the factors may vary depending on the structure [143,167]. As an example, the observed rate enhancement by the bimetallic complex **27b** and the mononuclear **28c** are equal, but inhibition studies by an unreactive substrate analog shows that while **27b** binds more strongly, **28c**, when bound, is more efficient as a catalyst (Figure 11) [167].

**Figure 12:**
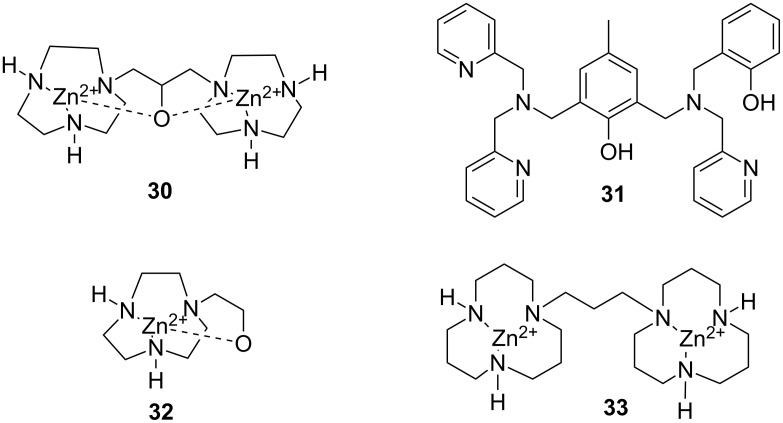
Miscellaneous complexes and ligands used in kinetic studies of metal-ion-promoted cleavage of nucleic acids.

The most intensively studied bimetallic catalysts for the cleavage of RNA models are **30** (Figure 12) and **27a** introduced by Morrow [166] and Williams [145], respectively. Complex **30** at 2 mmol L^−1^ concentration reduces the half-life of the cleavage of UpU to about one week at pH 7.0 and 25 °C [174] and **27a** is even more efficient: the half-life of UpU cleavage is only seven hours in the presence of 1 mmol L^−1^ **27a** at pH 6.5 and 25 °C [175]. **27a** and its Co^2+^ analog are unique among metal ion catalysts in that they modestly enhance also the interconversion of 3´,5´- and 2´,5´-dinucleoside monophosphates [175–176]. Catalysis on the hydrolysis of DNA models by these complexes has not been studied or is less significant than in the case of RNA models. Interestingly, very fast cleavage of highly activated DNA analog, bis(2,4-dinitrophenyl phosphate) (BDNPP; **23b**), has been observed in the presence of Tb^3+^, Eu^3+^ and Gd^3+^ complexes of ligand **31** in water/acetonitrile mixtures. Half-life less than 1 second has been reported for Eu^3+^-**31** at 1 mmol L^−1^ concentration at pH 7.0 and 25 °C [144]. The rate-enhancement compared to the background reaction is approximately 10^6^-fold. Larger non-enzymatic rate-enhancing effects have been obtained only in anhydrous methanol and ethanol with HPNP and its analog as substrates [177]. Kinetic data obtained with bifunctional catalysts is collected in Table 3.

**Table 3:** Pseudo first-order rate constants (s^−1^) of phosphoester hydrolysis/transesterification in the presence of bimetallic and monometallic complexes (1 mmol L^−1^) under neutral conditions.

Catalyst	HPNP	NpOAr	NpN

**32**	1.3∙10^−6^ (pH 7,61)^a^		
**30**	2.5∙10^−4^ (pH 7,61)^a^	0.1 (pH 7.5)^b^ NPP	9.9∙10^−7^ (pH 7.4)^c^
**28c**	4.6∙10^−5^ (pH 7.4)^d^		
**27a**	5.3∙10^−2^ (pH 7,4)^e^		2.6∙10^−5^ (pH 6.5)^f^

^a^From ref. [178]. Calculated from the second-order rate constant determined as the slope of *k*_obs_ vs *c*(complex) plot. ^b^From ref. [179]. Calculated from the second-order rate constant estimated from Figure 1. ^c^From ref. [174]. Calculated from the second-order rate constant determined as *k*_2_ = *k*_cat_/*K*_m_*. *^d^From ref*.* [167]. Calculated from the second-order rate constant determined as the slope of *k*_obs_ vs c(complex) plot. ^e^From ref. [168]. Calculated from the second-order rate constant determined as *k*_2_ = *k*_cat_/*K*_m_*.* Second-order rate constants determined as the slope of *k*_obs_ vs c(complex) plot. ^f^Observed pseudo first-order rate constants from ref. [175].

Even though many metal ion catalysts promote the cleavage of phosphodiester bonds, **27a** is the only catalyst that is known to enhance the mutual 3´,5´- to 2´,5´ isomerization of RNA phosphodiester bonds [175–176]. As discussed in the foregoing, isomerization is the predominant reaction of dinucleoside monophosphates and related nucleoside 3´-alkyl phosphates with a poor leaving group in the absence of metal ion catalysts at pH < 7, whereas activated phosphodiesters are not isomerized. There are two obvious reasons for the lack of isomerization in the presence of metal ion catalysts. Firstly, when the phosphorane intermediate obtained is dianionic, it is too unstable to pseudorotate. Evidently metal ion binding does not sufficiently stabilize the intermediate, or it retards pseudorotation. Alternatively, the departure of the leaving group by the exocyclic fission may be so efficiently enhanced that isomerization via the endocyclic cleavage cannot compete with it. The first step of the reaction may become rate-limiting or the reaction becomes a concerted process.

The catalysis of phosphate migration by **27a** is modest in comparison to the cleavage reaction. At a concentration of 1 mmol L^−1^ **27a** promotes the isomerization of UpU by a factor of 150, while the cleavage is accelerated up to 10^6^-fold [175–176]. Studies with a non-cleavable phosphonate analog have, however, verified the rate-acceleration of isomerization. Evidently, **27a** and its Co^2+^ and Cu^2+^ analogs stabilize the phosphorane to such an extent that pseudorotation can take place, probably through multiple interactions between the catalyst and the phosphorane. Consistent with this assumption, thiophilic Zn^2+^ accelerates the isomerization of phosphoromonothioate analog of UpU, although again the acceleration of isomerization is modest compared to the acceleration of cleavage, at [Zn^2+^] = 5 mM 6.4- and 410-fold, respectively [180].

#### Parameters describing the catalytic activity

The rate enhancing effects of metal ion catalysts can be described in several different ways that may give a different impression on the catalytic power of a given complex. A straightforward way to describe the efficiency of a metal ion catalyst is to give the ratio of pseudo first-order rate constants obtained in the presence and in the absence of the catalyst, as done in Table 2. Problems may, however, arise when the background reaction is slow. Rate constants under neutral conditions often have to be estimated by linear extrapolation from the rate constants measured under alkaline conditions without knowing whether the logarithmic rate constant really is linearly related to pH over the wide pH range employed. One should bear in mind that the shape of the pH-rate profile depends on polar nature of the leaving group [48,181]. Likewise, comparison between rate constants determined at different pH and catalyst concentration may easily lead to errors, if experimental data on dependence of rate on catalyst concentration at various pH values is not available, which very often is the case. In summary, care should be exercised on comparing the catalytic efficiencies of various catalysts.

Michaelis–Menten kinetics (Equation 1) has often been applied to metal-ion-catalyzed cleavage, particularly the cleavage of HPNP [143,145,182–183]. Parameters *K*_m_ (in mol L^−1^) and *k*_cat_ (in s^−1^) are the dissociation constants of the catalyst-substrate complex and the first-order rate constants for the breakdown of the catalyst-substrate complex to products. [S]_0_ and [catalyst]_0_ stand for the initial concentrations of the substrate and catalyst. The ratio *k*_cat_/*K*_m_, hence, is the measure of catalytic efficiency. This ratio actually is equal to the second order rate constant for the metal ion catalytic reaction, i.e., the slope of *k*_obs_ vs [catalyst] plot.

[1]
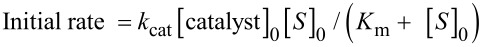


The ratio of *k*_cat_/*k*_0_*,* where *k*_0_ is the first-order rate constant for the uncatalyzed reaction, is sometimes used to describe the efficiency of a given catalyst. Values thus obtained are impressive, but may give an unrealistic impression, as *k*_cat_ refers to situation where all the substrate molecules are quantitatively bound to the catalyst; a situation that is rarely achieved. Comparison of *k*_cat_/*K*_m_ values shown in Table 4 puts the catalytic activity of even the most efficient metal ion catalysts into perspective. It can be seen that while the rate-enhancement obtained by bimetallic complexes is fairly impressive, it still falls far behind the catalytic activity of enzymes. Although the *K*_m_ term referring to the substrate binding is of the same order, the *k*_cat_ are several orders of magnitude smaller than those for enzyme catalysis. Sometimes catalytic activity is expressed as kinetic effective molarity that is defined as the ratio between the first-order rate constant of an intracomplex reaction and the second-order rate constant of the corresponding intermolecular reaction.

**Table 4:** Kinetic parameters for the catalysis of the HPNP cleavage by bimetallic complexes. Experimental details are described in the text.

catalyst	substrate	*k*_cat_ / s^−1^	*K*_m_ / mol L^−1^	[*k*_cat_*/K*_m_] / L mol^−1^ s^−1^ (= *k*_2_)

**27a**^a^	HPNP	0.017	3.2·10^−3^	53
**30**^b^	HPNP	4.1·10^−3^	0.016	0.25
**33** + 1 equiv MeO^−^ in MeOH^c^	HPNP			2.75·10^5^
**33** + 1 equiv MeO^−^ in MeOH^c^	BNPP	0.041	0.37·10^−3^	111
Tb^3+^-**31**^d^	BDNPP	18	0.006^e^	3000
RNase A^f^	HPNP	7.9·10^2^	7.9·10^−3^	1.0·10^5^

^a^From ref. [145]; ^b^from ref. [178]; ^c^from ref. [177]; ^d^from ref. [144]. Data refer to 75% MeCN in water; ^e^given as *K*_1_ = 166 mol^−1^ L (= 1/*K*_m_); ^f^ref. [184].

**Figure 13:**
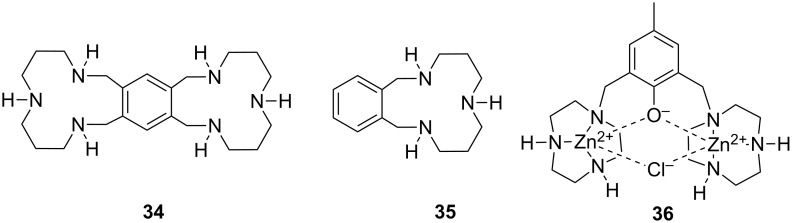
Azacrown ligands **34** and **35** and dinuclear Zn^2+^ complex **36** used in kinetic studies of metal-ion-promoted cleavage of nucleic acids.

As mentioned above, catalytic efficiency may be expressed by *k*_cat_/*K*_m_. Accordingly, it is of interest to understand to what extent each of these parameters contribute to the observed catalytic effect of various metal-based catalysts. Metal aqua ions and simple metal ion complexes generally bind monoanionic phosphodiesters only weakly. A frequently applied method to estimate the *K*_m_ value is inhibition of the cleavage with an unreactive structural analog of the substrate that binds to the metal ion catalyst approximately as tightly at the substrate [178]. Usually, HPNP is used as the substrate and dimethyl or diethyl phosphate as the inhibitor. The *K*_i_ values, dissociation constants of the catalyst–inhibitor complex, are then assumed to correlate with the *K*_m_ values. According to these studies, bifunctional catalysts generally bind to the inhibitor more strongly than their monomeric counterparts. Complexes **26a**,**b**, **27a**,**b** and **28c** offer an illustrative example of the stabilizing effect of increasing number of functional groups. The monomeric Zn^2+^ complex **26b** binds considerably less readily, *K*_i_ = 0.13 mol L^−1^, than its amino substituted analog **26a**, *K*_i_ = 0.01 mol L^−1^ [168]. Monomeric complex **28c** binds surprisingly weakly (*K*_i_ = 0.15 mol L^−1^), but the corresponding dimer, **27b**, binds much more tightly (*K*_i_ = 0.009 mol L^−1^) [167]. Additional amino groups still increase the affinity; the *K*_i_ value for **27a** is 0.32 mmol L^−1^ [145]. Likewise, the dinuclear Zn^2+^ complex of **34** (Figure 13) binds more tightly than the mononuclear Zn^2+^ complex of **35**, the *K*_m_ values being 0.007 mol L^−1^ and 0.0184 mol L^−1^, respectively [183]. One should, however, bear in mind that the structure of substrate may also play a role. For instance, dependence of the cleavage rate of BDNPP (**23b**) and HPNP (**1**) on concentration of **36** suggests that binding to BDNPP is weaker than binding to HPNP [182].

Nucleic acid bases offer additional potential coordination sites for metal ion complexes, resulting in tighter substrate binding. Uracil and guanine bases, in particular, are potential coordination sites as they undergo deprotonation around pH 9. Interaction of **30** with uracil bases has been suggested to be fairly strong [174]. According to kinetic inhibition studies, UpU is recognized almost one order of magnitude more efficiently than HPNP. In addition, uridine has been shown to inhibit the cleavage of HPNP promoted by Zn^2+^-polyazamacrocycle complexes [185].

#### pH-Rate profiles

Determination of pH-rate profile is very often the first experiment employed to study the mechanism of a reaction. Plots of *k*_obs_ (or *k*_2_ = *k*_obs_/[catalyst]) against pH are generally sigmoidal [151,159,182] or bell-shaped [162,169,172,186–187] for metal-ion-promoted reactions, independently of the type of substrate. Sigmoidal profile has been attributed to a catalyst with one dissociable functional group, whereas a bell-shaped profile has been taken as an indication of two such groups [160]. p*K*_a_ values determined on the basis of pH-rate profiles usually agree well with the values obtained potentiometrically for the catalyst complexes [159,168]. These results are often interpreted as an indication of the mono-deprotonated complex being the active catalyst and a metal-bound hydroxy or alkoxy group being involved in the reaction. Consistent with this, metal complexes with lowest p*K*_a_ values are usually the most efficient catalysts at a fixed pH [148,188].

The descending part of a bell-shaped pH-rate profile has been taken as an indication of a second deprotonation that renders the catalyst inactive. Most logical explanation for the inactivation is release of the substrate: the hydroxide ion and the substrate compete for the metal ion and at sufficiently high concentration of hydroxide ions the binding starts to weaken [162,168]. With a multifunctional catalyst, the decreasing catalytic activity may also result from deprotonation of a functional group directly involved in the catalysis. A third factor, rarely considered in this context, is decreasing stability of the catalyst complex. Formation of precipitates is sometimes observed at higher pH’s [166,183], but inactivation of the catalyst may take place already before visible precipitation. Reaction time is also crucial; complexes that are efficient catalysts in reactions of HPNP over a wide pH-range may become inactivated on a time scale required to follow reactions of non-activated substrates.

Another fact that complicates the mechanistic interpretations on the basis of pH-rate profiles is that the background reaction usually is base-catalyzed. Even though the observed first-order or second-order rate constants increase upon increasing pH, the catalytic activity of metal ion complexes may actually decrease. This is clearly seen with the pH-rate profile reported for HPNP cleavage promoted by **27b** [167]. In addition, when quantitative data on the pH-dependence of binding equilibrium is not available, the concentration of catalyst–substrate complex at a given pH is not known and, hence, the reaction system is not accurately defined. Despite the shortcomings discussed above, it is clear that deprotonation at pH close to p*K*_a_ of a metal bound aqua ligand plays a significant role in catalysis and it often serves as the basis of mechanistic conclusions.

#### Effect of substrate structure; β_lg_ values

The results in Table 2 show that the rate-enhancement observed for three RNA models, viz. HPNP, nucleoside 3´-(*p*-nitrophenyl phosphate) and dinucleoside-3´,5´-monophosphate, are within the same magnitude, the largest values being more often obtained with HPNP. There is one clear exception: with Cu^2+^-Terpy the largest rate-enhancement is obtained with a dinucleoside-3´,5´-monophosphate or nucleoside 3´-alkyl phosphate with an equally poor leaving group. This may possibly be attributed to dimerization of Cu^2+^-TerPy under the experimental conditions; different substrates seem to respond differently in dimer formation [153].

**Figure 14:**
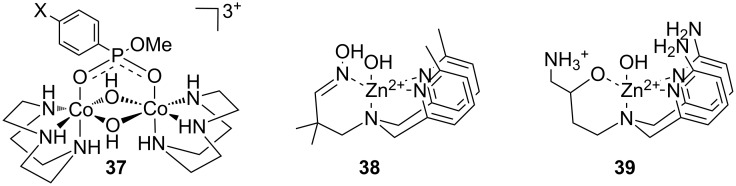
Metal ion complexes used for determination of β_lg_ values of metal-ion-promoted cleavage of RNA model compounds.

Despite the apparent similarity of the overall influences, differences in the behavior between alkyl and aryl esters are accounted when the susceptibility to the polar nature of the leaving group is considered [189]. β_lg_ values collected in Table 5 show that there are differences between different types of catalysts (Figure 14) as well as between substrates. Values obtained with nucleoside alkyl esters are generally modestly negative on using metal aqua ions as a catalyst [149,176,189–190]. In this respect, the reaction resembles acid-catalyzed transesterification of nucleoside phosphodiesters [49], and the similarity has been taken as an indication of protonation of the leaving group in the rate-limiting step [190]. β_lg_ values obtained with Ni^2+^ or metal ion complexes are slightly more negative than that obtained with Zn^2+^, but they still are clearly less negative than the value reported for the alkaline cleavage, viz. −1.28 at 90 °C [49]. The values evidently reflect varying degree of protonation that, in turn, depends on the acidity of aqua ligand of the complex and the coordination geometry around the metal cation. The fairly negative value of −0.92 obtained in the presence of 1 mmol L^−1^ **27a** has been compared [176] to the value, −0.94, reported for the pH-independent reaction of nucleoside 3´-(dialkyl phosphate)s [50]. In the latter reaction the leaving group departs as alcohol with concerted proton transfer from a general acid.

**Table 5:** β_lg_ values for cleavage reactions of phosphodiesters promoted by metal ion catalysts.

substrate	catalyst / conditions	β_lg_	ref.

MePAr	**37**	−1.38 ± 0.01	[193]
MePAr	**38**	−1.2 ± 0.1	[187]
3´-UMP aryl esters	10 mmol L^−1^ Zn(NO_3_)_2_, pH 5.9, 25 °C	−0.9 ± 0.2	[190]
3´-UMP aryl esters	10 mmol L^−1^ Zn-TACD, pH 7.5, 25 °C	−0.81 ± 0.07	[189]
3´-UMP alkyl esters	10 mmol L^−1^ Zn(NO_3_)_2_, pH 5.6, 90 °C	−0.32 ± 0.04	[190]
3´-UMP alkyl esters	2 mmol L^−1^ ZnCl_2_, pH 5.6, 90 °C	−0.36 ± 0.02	[176]
3´-UMP alkyl esters	10 mmol L^−1^ NiNO_3_, pH 5.6, 90 °C	−0.54 ± 0.03	[191]
3´-UMP alkyl esters	10 mmol L^−1^ Zn-TACD, pH 6.6, 90 °C	−0.6 ± 0.1	[191]
3´-UMP alkyl esters	2 mmol L^−1^ Zn-TACN, pH 6.6, 90 °C	−0.51 ± 0.04	[191]
3´-UMP alkyl esters	2 mmol L^−1^ Zn-cyclen, pH 6.6, 90 °C	−0.71 ± 0.06	[191]
3´-UMP alkyl esters	2 mmol L^−1^ Ni-TACD, pH 6.6, 90 °C	−0.58 ± 0.04	[191]
3´-UMP alkyl esters	1 mmol L^−1^ **27a**	−0.92 ± 0.07	[176]

The β_lg_ values of the cleavage of aryl esters are more negative than those obtained with nucleoside alkyl esters [149,176,189,191], typically around −0.9. They are also more negative than the values obtained in the absence of metal ion catalysts, −0.58 [192] and −0.54 [51] for the hydroxide-ion-catalyzed cleavage of alkyl aryl phosphates and nucleoside aryl phosphates, respectively. In the case of the Co^3+^-complex-promoted cleavage of alkyl aryl phosphates, the markedly negative β_lg_ has been attributed to significant bond strain, resulting from a formation of a four-membered ring upon nucleophilic attack of the bridging hydroxo ligand on phosphorus [192]. As regards nucleoside aryl phosphates, the most logical explanation is that metal ion binding stabilize the phosphorane intermediate and, hence, shifts the transition state towards the products obtained by departure of aryloxy anions. In other words, the concerted mechanism with rate-limiting formation of the phosphorane that operates in the absence of a metal ion catalyst is altered towards a stepwise mechanism. In summary, with nucleoside aryl phosphates, the metal-ion-promoted cleavage is more sensitive than the background reaction to the electronegativity of the leaving group (−0.9 vs −0.5), whereas with alkyl phosphates the situation is the opposite (−0.5 vs −1.3). This essentially means that the rate-enhancing effect of metal ions, when expressed as *k*_cat_/*k*_0_, increases when an aryl leaving group becomes better or an alkyl leaving group becomes poorer [189].

The use of dinucleoside-3´,5´-monophosphates as model compounds brings about an additional feature not present in simpler model compounds; two nucleic acid bases provide additional binding sites for catalysts. Catalysis by monometallic species is fairly insensitive to the base composition: rate constants of 15 different dinucleoside monophosphates differed within a factor of two in the presence of 10 mmol L^−1^ Zn^2+^ at pH 5.1 and 90 °C [194]. In contrast, catalysis by Cu^2+^-TerPy is markedly base moiety selective: among four dinucleoside monophosphates studied, an 8-fold difference was observed between the most (ApA) and least (UpU) reactive substrates [165]. With more complex catalysts, the differences can be even larger: a 500-fold reactivity difference has been reported for a trinuclear calix[4]arene-based Cu^2+^ catalyst, UpU and CpA being the most and least reactive, respectively [155]. Bifunctionalized calix[4]arene bearing Cu^2+^-TACN and a guanidinium group also show marked selectivity. GpA is 130 times more reactive than CpA [171]. A dimeric catalyst with two Cu^2+^-TerPy units favors, in turn, ApA as the substrate [165]. In contrast to these results, rate-enhancement by **27a** is fairly insensitive to base composition: among five different 3,5-dinucleoside monophosphates studied, only a 3.5-fold difference was observed [176]. Preferred binding of Zn^2+^ azacrown chelates to uracil has been exploited in developing di- and trinuclear base moiety selective cleaving agents for RNA [195–196].

#### Heavy atom and solvent isotope effects

Heavy atom isotope effects lend further support for the view that the transition state of metal-ion-promoted cleavage of RNA is late compared to the hydroxide-ion-catalyzed cleavage (Table 6). While the ^18^*k*_lg_ value for specific base-catalyzed cleavage of UpG is 1.0343, the same isotope effect for the Zn^2+^-promoted reaction is 1.015, still normal but considerably smaller and, hence, consistent with more rigid bonding to the leaving group [197]. The ^18^O isotope effect for the attacking nucleophile is inverse for the metal-ion-catalyzed reaction, ^18^*k*_nuc_ = 0.986. The values are consistent with a late transition state, with significant bond formation between the nucleophile and phosphorous [197]. When dinuclear Zn^2+^ complex **30** is used as a catalyst and HPNP as a substrate ^18^*k*_lg_ = 1.0113 and ^18^*k*_nuc_ = 0.9874 [198]. The values closely resemble those obtained with UpG and differ more markedly from those of the hydroxide-ion-catalyzed cleavage of HPNP. Accordingly, Zn^2+^-promoted cleavage of both UpG and HPNP appears to proceed via a similar late transition state, whereas mechanisms of the hydroxide-ion-catalyzed reactions are different: HPNP is cleaved by rate limiting formation and UpG by rate limiting breakdown of the phosphorane intermediate.

**Table 6:** Heavy-isotope effects determined in the presence and absence of metal ion catalysts.

catalyst	substrate	^18^*k*_nuc_	^18^*k*_lg_	^18^*k*_NB_	^15^*k*_NO2_	ref.

**30**^a^	HPNP (**1**)	0.9874^b^	1.0113		1.0015	[198]
HO^− c^	HPNP (**1**)	1.0079^b^	1.0064		1.0002	[198]
**30**	HPNP (**1**)	0.9926^d^	1.0042^d^			[200]
H_2_O	HPNP (**1**)	1.0182^d^	1.0021^d^			[200]
Zn^2+ e^	UpG	0.986	1.015	1.0007		[197]
HO^− f^	UpG	0.997	1.0343	0.999		[197]
CuTACN^g^	EtPNP^h^				1.0013	[199]
HO^−^	EtpNP				1.0016	[199]

^a^pH 7.8 HEPES buffer, 40 °C. ^b^Observed values have been corrected for the calculated EIE for deprotonation of HPNP. ^c^pH 10.1 CHES buffer, 67 °C. ^d^Based on DFT calculation. ^e^10 mmol L^−1^ ZnNO_3_, pH 7, 90 °C; ^f^pH 12, 90 °C; ^g^pH 7.2, 70 °C, ^h^ethyl *p*-nitrophenyl phosphate.

The secondary ^15^N isotope effect (^15^*k*) for the nitro group of *p*-nitrophenol leaving group is particularly useful, for it can be regarded as a measure of the charge development on the leaving group oxygen. The value of 1.0013 observed for the Cu^2+^-TACN-promoted reaction of ethyl *p-*nitrophenyl phosphate (EtPNP) has been attributed to 46% bond cleavage in the transition state [200]. A value of the same magnitude has been observed for the transesterification of HPNP-promoted by **30** [194]. The value of 1.0002 for the specific base-catalyzed reaction has been considered insignificant and consistent with reaction where the formation of the phosphorane is rate-limiting.

The kinetic solvent isotope effect (KSIE), in turn, shed light to any kinetically significant proton transfer that occurs in a pre-equilibrium or rate-limiting step. In case no KSIE is observed, no proton transfer takes place. *k*_H_/*k*_D_ values close to unity are generally considered as an indication of a nucleophilic mechanism. In practice, the interpretation of the results is much more complicated, for the total effect observed may consist of opposing contributions. For example, an inverse equilibrium isotope effect (EIE) on deprotonation of a metal bound L_2_O ligand (L is H or D in any combination) and a normal EIE on deprotonation of the attacking nucleophile may result in an observed KSIE close to unity. Interactions with hydrogen bonding groups may also contribute to the observed KSIE, a fact that is often ignored when KSIE values are interpreted, even in cases where such a group significantly enhances the catalytic activity under consideration (e.g., [170]).

Often conditions are chosen to avoid any ambiguity resulting from pre-equilibrium proton transfer in order to obtain a KSIE that refers to the catalytic step only. For example, the KSIE of 1.43 reported for the transesterification of HPNP has been determined at pH 10.5 that is well above the kinetic p*K*_a_ of the catalyst [159]. According to the authors, the nucleophile is totally deprotonated both in H_2_O and D_2_O. If this is the case, the KSIE reflects the nucleophilic attack that inevitably takes place in the reaction, but gives no information on how the reactive ionic form has been formed. In case a significant KSIE is observed at p*L* < p*K*_a_ of the catalyst but not at p*L* markedly higher than the p*K*_a_ of the catalyst, a proton transfer is involved in a pre-equilibrium process [169,201].

An exceptionally large KSIE of 13.2 has been reported for the transesterification of a dinucleoside monophosphate, UpG, in the presence Zn^2+^ [197]. There may be other contributing factors, such as interactions to nucleic acid bases, but a very likely explanation stems from precipitation of Zn^2+^ lyoxo species under the experimental conditions. Examples of KSIEs determined for metal-complex-promoted cleavage of DNA and RNA models are listed in Table 7.

**Table 7:** Solvent isotope effects reported for reactions of phosphodiesters in the presence of metal ion catalysts.

catalyst	substrate	conditions/reaction	KSIE	ref.

Cu^2+^-TACN	EtPNP^a^	pH 9	*k*_H_/*k*_D_ = 1.14	[199]
**39**	BNPP (**23a**)	catalysis by a mono-deprotonated species	*k*_2,H_/*k*_2_*_,_*_D_ = 0.8	[169]
**36**	BNPP (**23a**)	p*L* = 7.9	*k*_H_/*k*_D_ = 1.26	[182]
Tb^3+^-**31**	BDNPP (**23b**)	p*L* = 7, 75% MeCN	*k*_H_/*k*_D_ = 1.14	[144]
Cu^2+^-TerPy	cAMP^b^	catalysis by a mono-deprotonated species	*k*_2,H_/*k*_2_*_,_*_D_ = 1	[151]
**40**	HPNP (**1**)	pH 10.5	*k*_H_/*k*_D_ = 1.43	[159]
**36**	HPNP (**1**)	p*L* = 7.3	*k*_H_/*k*_D_ = 2.76	[182]
**30**	UpPNP (**24**)	p*L* > 9	*k*_c,H_/*k*_c_*_,_*_D_ = 0.8	[201]
Zn^2+^	UpEtoEt (**25**)	p*L* = 5.6, 90 °C	*k*_H_/*k*_D_ = 2.7	[176]
**27a**	UpEtoEt (**25**)	p*L* = 6.5, 25 °C	*k*_H_/*k*_D_ = 2.7	[176]
Zn^2+^	UpG	p*L* = 7.0, 90°C	*k*_H_/*k*_D_ = 13.2	[197]

^a^Ethyl *p*-nitrophenyl phosphate; ^b^adenosine 2´,3´-cyclic phosphate.

Zhang et al. [197] have additionally carried out proton inventory studies on Zn^2+^-promoted transterification of UpG. The curve *k_n_*/*k*_0_ vs isotopic ratio *n* was strikingly similar in shape to the one obtained for lyoxide-ion-catalyzed reaction. According to the authors, these curves were consistent with two normal fractionation factors: a large equilibrium effect due the deprotonation of the nucleophile, and another normal effect resulting from the solvation of the transition state.

#### Medium effects

**Figure 15:**
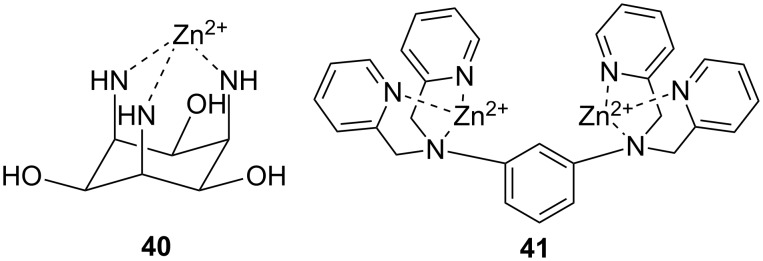
Metal ion complexes used in kinetic studies of medium effects on the cleavage of RNA model compounds.

The solvent composition may have a dramatic effect on the rate of metal-ion-complex-promoted reactions, either rate acceleration or deceleration. The most impressive rate-enhancing effect has been reported for the cleavage of activated phosphodiesters by the dinuclear Zn^2+^ complex **33** in the presence of 1 equiv of alkoxide ion in methanol [177] and ethanol [202]. Rate-enhancements up to 10^12^ in comparison to the corresponding background reactions have been observed with HPNP and methyl *p*-nitrophenyl phosphate (MePNP) in methanol [177,203]. In ethanol, the rate enhancement is even higher and the difference increases as the p*K*_a_ of the leaving group increases [202–203]. The significant rate enhancements result from stronger binding of the catalyst to substrate and from the reduced permittivity of the medium that allows closer contacts with and within the catalyst. Monomeric Zn^2+^-TACD complexes, for example, have been observed to act cooperatively at high concentration [177], in striking contrast to the behavior in water. Likewise, the dimeric catalyst **33** cleaves HPNP much more effectively than its monomeric counterpart in methanol but not in water [204]. Any structural change that expectedly weakens association, diminish the rate-enhancing effect of medium. Complex **41** (Figure 15) with a more rigid structure is clearly less efficient than **33**(MeO^−^) as a catalyst in methanol [205] and N*-*methylation of various azacrown-based complexes markedly decreases their catalytic efficiency in methanol [206].

Owing to very efficient cleavage of HPNP in the presence **33** (MeO^−^) in methanol, binding of the catalyst to substrate becomes rate limiting [205]. The efficiency of the binding events has been evaluated by using colored Cu^2+^ analog of **33** (MeO^−^) as a catalyst [207]. The colorimetric analysis showed that binding is a two-step process. The first of these is very fast and the rate is linearly dependent on the catalyst concentration. The second is a concentration-independent rearrangement that forms the active species with dinuclear Cu^2+^ coordination. The rate constants for the latter step are almost equal with MePNP and HPNP, 0.57 s^−1^ and 0.72 s^−1^, respectively. As the rate constant for the chemical cleavage of HPNP under the same conditions is 0.7 s^−1^, the latter binding step is rate-limiting. With the less reactive DNA analog, MePNP, the chemical cleavage step still is clearly rate-limiting.

In contrast to alcohols, DMSO and acetonitrile have been shown to retard the metal-complex-promoted cleavage of phosphodiesters. The effect of DMSO has been utilized to distinguish between general base-catalyzed and specific base-catalyzed reaction routes, as specific base-catalyzed reactions are suppressed in DMSO rich mixtures, owing to suppressed autoprotolysis of water [208]. Second order rate constants for the metal-ion-promoted reactions have been determined in 80% aqueous DMSO in different buffers keeping the buffer ratio constant but increasing the total buffer concentration. When the rate constants are plotted against the buffer ratio or the concentration of the base form, the shape of the plots indicates whether either a specific base or a general base-catalyzed reaction is suppressed. According to such an analysis, all metal ions studied enhance the specific base-catalyzed reaction of HPNP, whereas the general base-catalyzed reaction is assisted only by Mg^2+^ and Na^+^. KSIE values of 0.25 and 0.36 have been determined for the specific base-catalyzed reactions in the presence of Mg^2+^ and Ca^2+^, respectively and a value of 1.23 for the Mg^2+^-assisted general base-catalyzed reaction.

Despite the inhibition, organic co-solvents are often used to improve the solubility of the substrate or the catalyst [143–144,171]. In some cases the inhibition is strong enough to completely prevent the catalysis, although conflicting reports also exist. While Zn^2+^-TACD has been reported to catalyze the cleavage of HPNP efficiently in 50% aqueous acetonitrile [156], complete inactivation of Cu^2+^ and Zn^2+^ complexes of a related catalyst **35** was observed in the same medium [183]. The authors have speculated that the cyano group of acetonitrile binds the catalysts hence occupying one or more coordination sites of the catalysts.

#### Mechanistic conclusions

**Scheme 14:**
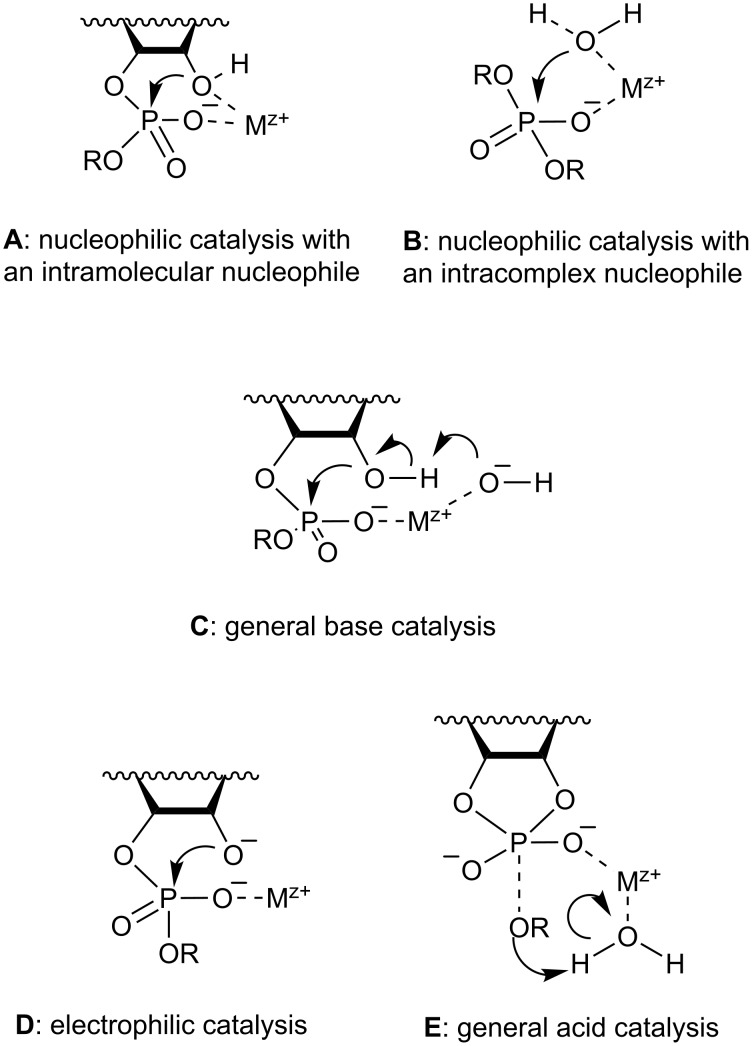
Alternative mechanisms for metal-ion-promoted cleavage of phosphodiesters.

Despite extensive studies, no universally accepted mechanism for metal ion catalysis has been found. There is, however, a fairly unanimous understanding of the importance of deprotonation event at pH close to that of the p*K*_a_ of a metal bound aqua ligand. Three different basic mechanisms have been proposed to explain the need for deprotonation: intracomplex nucleophilic catalysis (**A** and **B** in Scheme 14), intracomplex general base catalysis (**C**) and electrophilic (**D**) or general acid (**E**) catalysis on an intermediate obtained by a specific base-catalyzed reaction. Intermolecular general base or nucleophilic mechanisms are not considered feasible, since the catalysis by metal ion species is much more significant than by organic bases or nucleophiles.

The nucleophilic mechanism in this context involves a nucleophilic attack by a group coordinated to the metal ion catalyst. In case of DNA type substrates [159–160,182,198], the nucleophile is likely to be a metal bound hydroxo ligand (**B**), whereas with RNA type of substrates the nucleophile is the neighboring OH group on the substrate (**A**). Metal ion catalysts enhance deprotonation of the nucleophile by coordination, and since the p*K*_a_ values of metal-bound H_2_O and alcohols are likely to be of the same order of magnitude, the pH-dependence for reactions of both types of substrates is generally similar.

The nucleophilic mechanism is widely accepted for the reactions of DNA model compounds, such as BNPP [145,160,169,199], but also for HPNP [159–160]. KSIE values are close to unity, which is generally regarded as an evidence of a nucleophilic mechanism. Furthermore, it has been reported that under conditions where the metal-bound aqua ligand is completely deprotonated, the catalytic activity of metal ion catalysts increases with increasing p*K*_a_, as long as complexes of similar type (tridentate vs tetradentate) are concerned. This has been suggested to indicate that the catalytic activity at high pH depends on nucleophilicity of the metal-bound hydroxy ligand [159]. Tetradentate complexes are less efficient catalysts than tridentate ones of similar acidity, consistent with the need of a free hydroxo ligand to act as a nucleophile [160].

The dependence on the p*K*_a_ of the catalysts is similar in reactions of BNPP and HPNP, and the KSIE of 1.45 determined for the transtesterification of HPNP at pH 10.5 is within the range typical for nucleophilic catalysis [159]. In contrast, bimetallic complex **36** has been suggested to enhance the reaction BNPP by different mechanisms [182]. pH-Rate profiles for the reactions of the two substrates are different suggesting that different deprotonation events are involved. Furthermore, KSIE effects determined under the same conditions point to different mechanisms: while that for the reaction of BNPP is typical for nucleophilic catalysis, a value of 2.76 determined for the reaction of HPNP is of a magnitude typical to general base catalysis.

**Figure 16:**
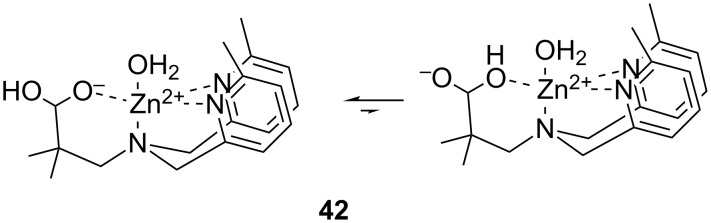
Nucleic acid cleaving agents where the attacking oxyanion is not coordinated to metal ion.

A metal-ion-bound hydroxide or alkoxide ion certainly is a weaker nucleophile than their free counterparts. Still virtually all metal-ion-based catalysts for the cleavage of BNPP are based on the attack of a metal-ion-bound nucleophile. Only rather recently, it has been shown that by carefully ligand design a situation may be achieved, where an unbound alkoxy group serves as a powerful nucleophile [209]. The key feature is a hydrated aldehyde group locked by a proper position Zn^2+^ coordinated additionally to three nitrogen atoms within ligand **42** (Figure 16). The *gem-*diol system may be coordinated to the central ion through alkoxy oxygen, but also through hydroxy oxygen, leaving the alkoxy function free to serve as a nucleophile. Although the latter species is a minor tautomer, its reactivity is high enough to overcome the unfavorable equilibrium.

Electrophilic catalysis or Lewis acid catalysis (**D**) has repeatedly been suggested for the reactions of RNA type substrates. The phosphate-bound metal ion catalyst activates the substrate towards nucleophilic attack, the nucleophile being neutral or deprotonated depending on the pH. The sigmoidal or bell-shaped pH-rate profiles can be understood by considering the effects of increasing pH on both the catalyst and substrate. The proportion of the anionic nucleophile, and hence, the efficiency of the nucleophilic attack is increased as long as the p*K*_a_ value of the secondary OH group around pH 12 is reached. On passing the p*K*_a_ value of catalyst aqua ligands, generally at pH 7–9, binding to the phosphodiester group is weakened and the electrophilic contribution of the catalysis is lost. This mechanism has been proposed to be utilized, for example, by the most efficient bifunctional catalysts **30** and **27a** of the transesterification of HPNP. Williams et al. [168] have justified their mechanistic choice by studying the two kinetically equivalent mechanisms: deprotonation of neutral substrate by a deprotonated complex that acts a general base, and specific base-catalyzed reaction of a substrate activated by the aqua form of the catalyst. Because dimethyl phosphate inhibits the reaction more strongly at a lower pH, where the proportion of the aqua form is higher, it has been concluded that the inhibitor competes for the aqua form. This has been taken as an evidence of the electrophilic mechanism, where the aqua form is the active catalyst (**D**).

Transesterification of nucleoside phosphoesters, UpNP and UpU, by **30** has also been suggested to proceed by mere electrophilic catalyzed pathway [170]. Similar pH-dependence with three different types of substrates has been taken as an indication of similar ionic forms being important in the reactions. Furthermore, a KSIE value of 0.8 has been determined for **30**-promoted reaction of UpNP at p*L* > 9, which shows that no proton transfer takes place in the reaction, when the formation of the phosphorane is rate-limiting (**D**). A proton transfer to assist the departure of the poor leaving group of UpU has been rejected on the basis of microscopic reversibility.

In contrast to catalysis by **30**, two different mechanisms have been proposed for the **27a**-promoted reactions of HPNP and UpU. While HPNP with a good leaving group is most probably cleaved without general acid/base catalysis (**D**) [167], a KSIE of 2.7 for the **27a**-catalyzed reaction of UpEtOEt with a poor leaving group suggests proton transfer in the rate-limiting step [176]. Since the KSIE for the **27a**-promoted phosphate migration is close to unity [176], the proton transfer most probably enhances the breakdown of the phosphorane intermediate to cleavage products. In other words, general acid catalysis appears to be involved (**E**).

General base catalysis by a metal-bound hydroxo ligand (**C**) is the most obvious way of interpreting the sigmoidal or bell-shaped pH-rate profiles**.** The rate of reaction increases as the proportion of the hydroxo form of the catalyst is increased. At pH < p*K*_a_ of the metal-bound aqua ligands, the hydroxo form of the catalyst is the strongest base. KSIE values determined under such conditions fall within the range usually attributed to general base catalysis [176,182]. It has, however, been recognized that a kinetically equivalent specific base – general acid catalysis, i.e., pre-equilibrium deprotonation of the attacking nucleophile followed by general acid-catalyzed breakdown of the intermediate, appears more feasible when the substrate has a poor leaving group. Consistent with this suggestion, modestly negative β_lg_ values have been observed for metal-ion-promoted reactions of nucleoside 3’-alkyl phosphates [176,190]. In this respect, metal-ion-promoted reactions resemble more the acid-catalyzed reaction than the base-catalyzed. Furthermore, an analysis of the effect of the acidity of the leaving group alcohol on the catalysis by various metal ion complexes shows that the most acidic catalysts fail to promote the transesterification of the substrates with most basic leaving groups [189]. Results obtained with ^18^O experiments on Zn^2+^-catalyzed reaction of UpG [197] may also be taken as an indication of catalysis mechanism that affects the departure of the leaving group.

The preceding discussion shows that all three basic mechanistic alternatives are firmly supported by experimental evidence. Theoretical calculations based on density functional theory do not solve the controversy, either [160,200,210]. All theoretical studies generally support a concerted reaction mechanism and indicate a number of important interactions to the nucleophile, phosphate and leaving group. Many of the studies concentrate also on the deprotonation of the nucleophile and both pre-equilibrium [200,211] and concerted processes [183,210] have been predicted. Regardless of timing, the nucleophile may also be coordinated to a metal ion [160,200,210].

Most probably the mechanism depends on both the substrate and the catalyst. Consistent with this, there are examples showing that two different types of substrates may be cleaved by two different mechanisms in the presence of the same catalyst. Furthermore, an analysis of the effect of the acidity of the leaving group in nucleoside phosphodiesters shows, that, generally, a more efficient catalysis is observed when there is an imbalance between the properties of a nucleophile and the leaving group. Results in Table 2 suggest that this may be extended even further and the extremes on the scale would be DNA model BNPP with no intramolecular nucleophile and a good leaving group, and dinucleoside monophosphates with a favorably positioned nucleophile and a poor leaving group. It would seem logical to assume that catalysis required in corresponding reactions is different.

Beyond the scope of the present review are the nanostructured cleaving agents that show cooperativity between the catalytic functions on particle surface [212] and sequence-selective cleaving agents that consist of an artificial cleaving agent conjugated to a sequence recognizing moiety [213]. Finally, it is worth noting that in spite of extensive studies of the metal-ion-promoted cleavage of nucleic acids, the applications still are scanty. There is only one patiently developed application that deserves to be highlighted, viz. the manipulation of large genomes by Ce^4+^-promoted cleavage followed by enzymatic ligation. The description of this fascinating technique is, however, outside the scope of the present paper. Recent reviews on the subject [214–216] are recommended.

## Conclusion

Experimental studies with small molecular model compounds of nucleic acids allow evaluation of the importance of various elementary processes, such as proton transfer and metal ion binding, for stabilization of transition states and systematic variation of the basicity of the entering and departing nucleophile enables determination of the position of transition state on the reaction coordinate. Such data is helpful on analyzing mechanisms of enzymatic processes. Studies with RNA models have been more extensive than those with DNA models. The predominant buffer-independent reactions of RNA 3’,5’-phosphodiester linkages under neutral conditions are approximately as fast pH-independent isomerization to 2´,5´-bonds and hydroxide-ion-catalyzed transesterification to a 2´,3´-cyclic phosphate. The kinetics and mechanisms of these reactions are rather well known. By contrast, the detailed mechanisms of buffer-catalyzed reactions still seem to be open to various interpretations of kinetic data. Catalysis by multifunctional agents containing amino, imidazole and guanidine groups have received special attention, owing to presence of such functions at the side chains of catalytically important amino acids in nucleases. The mechanistic studies on cleavage of DNA are scanty. The very high stability of the phosphodiester bonds within DNA has clearly limited the interest. The metal-ion-promoted cleavage of both RNA and DNA has recently received increasing interest. Extensive studies have led to a number of mechanistic suggestions, but more systematic studies with various substrates and catalysts are still needed to draw firm mechanistic conclusions.
